# Efficacy and safety of abaloparatide, denosumab, teriparatide, oral bisphosphonates, and intravenous bisphosphonates in the treatment of postmenopausal osteoporosis: a systematic review and Bayesian network meta-analysis

**DOI:** 10.3389/fendo.2026.1886136

**Published:** 2026-07-09

**Authors:** Yan Wang, Xiaoyan Wang, Guihong Yu, Libao Zhang, Changhui Xue, Wanchen Gong, Jiashou Luo, Chengwu Lu, Linfeng Wang

**Affiliations:** 1 Department of Orthopedics, Nanping First Hospital Affiliated to Fujian Medical University, Nanping, China; 2 Department of Gynaecology and Obstetrics, Jian’ou Maternal and Child Health Hospital, Nanping, China; 3 Fujian Minbei Orthopedics Research Institute, Nanping, China

**Keywords:** abaloparatide, Bayesian network meta-analysis, bisphosphonates, postmenopausal osteoporosis, teriparatide

## Abstract

**Background:**

Postmenopausal osteoporosis increases the risk of fractures, particularly in the lumbar spine, femoral neck, and total hip. Pharmacological interventions include anabolic agents (abaloparatide [ABA], teriparatide [TER]), antiresorptive agents (oral/intravenous bisphosphonates [OBP/IBP], denosumab [DEN]), and conventional therapy or placebo (PLA/CTRL). Evidence on their comparative efficacy and safety in postmenopausal women is limited.

**Methods:**

We conducted a Bayesian network meta-analysis of randomized controlled trials (RCTs) to evaluate the relative efficacy and safety of ABA, TER, OBP, IBP, DEN, and PLA/CTRL in postmenopausal women with osteoporosis. Primary outcomes included changes in lumbar spine, femoral neck, and total hip bone mineral density (BMD). Safety outcomes included all adverse events (AEs) and serious adverse events (SAEs). Data were synthesized using SUCRA rankings and network consistency was assessed via node-splitting and deviance information criteria (DIC).

**Results:**

Twenty-three RCTs comprising 80–7, 808 postmenopausal women were included. ABA demonstrated the greatest improvement in lumbar spine and femoral neck BMD, followed by TER, while ABA showed the highest effect on total hip BMD. IBP and DEN provided moderate benefits, superior to PLA/CTRL. TER and OBP had the lowest risk for AEs, and ABA and PLA/CTRL showed the lowest risk for SAEs. Overall, anabolic agents significantly improved BMD at the spine and femoral neck, whereas antiresorptive agents were more effective for hip BMD.

**Conclusions:**

ABA and TER are most effective for improving spinal and femoral neck BMD in postmenopausal women, whereas ABA is superior for total hip BMD. Safety profiles were favorable for TER, OBP, and ABA. These findings provide evidence to guide individualized treatment selection based on fracture risk and high-risk skeletal sites in postmenopausal women.

## 1 Introduction

Osteoporosis is a systemic skeletal disorder characterized by decreased bone mass and deterioration of bone microarchitecture, resulting in increased fracture risk, particularly among postmenopausal women (1). The decline in estrogen levels following menopause exacerbates bone metabolism imbalance, leading to insufficient bone formation and excessive bone resorption, which increases the risk of fractures in the spine, hip, and other skeletal sites (2). The lumbar spine, femoral neck, and total hip are common high-risk fracture sites, and changes in bone mineral density (BMD) at these sites not only influence fracture incidence but also directly affect patients’ quality of life and long-term health outcomes. Therefore, effective preservation and improvement of bone mass at these critical sites are central goals in managing postmenopausal osteoporosis (3).

Pharmacologic treatments for postmenopausal osteoporosis primarily include anabolic agents (e.g., abaloparatide [ABA], teriparatide [TER]), antiresorptive agents (e.g., oral/intravenous bisphosphonates [OBP/IBP], denosumab [DEN]), and placebo or conventional therapy (PLA/CTRL) (4–6). These agents differ in their effects on bone density, fracture prevention, and safety profiles. Anabolic agents stimulate osteoblast activity to increase bone mass, whereas antiresorptive agents primarily inhibit osteoclast activity to reduce bone resorption, leading to site-specific variations in treatment efficacy (7, 8). However, existing comparative studies in postmenopausal women are limited, often involving only pairwise comparisons or small sample sizes, making it challenging to provide comprehensive, quantitative evidence for clinical decision-making (9, 10). Recently, several systematic reviews have further refined the evidence base for anabolic therapy in osteoporosis. Bonifacio et al. (11) systematically evaluated the efficacy and safety of abaloparatide and reported that abaloparatide was associated with substantial improvements in lumbar spine BMD and reductions in vertebral fracture risk, while its effects on hip and femoral neck BMD were comparatively smaller and more heterogeneous. In addition, Cipolloni et al. (12) assessed the anabolic-first strategy in patients at very high fracture risk and suggested that initiating therapy with anabolic agents may provide faster and more pronounced fracture-risk reduction than antiresorptive-first approaches. These studies highlight the growing importance of anabolic agents in osteoporosis management. However, they primarily focused on abaloparatide-specific evidence or fracture outcomes in very high-risk populations, whereas comparative evidence across multiple pharmacological classes and skeletal sites in postmenopausal women remains insufficiently characterized. Therefore, a network meta-analysis comparing anabolic agents, antiresorptive agents, and placebo/conventional therapy may provide additional evidence for individualized treatment selection.

Network meta-analysis (NMA) can integrate both direct and indirect comparisons to systematically evaluate multiple interventions and rank their relative efficacy, providing high-level evidence to guide clinical decisions. Although NMAs have been conducted in male or mixed-sex populations, postmenopausal women may exhibit distinct pharmacological responses due to hormonal changes and bone metabolism characteristics (13–15).

Therefore, this study aimed to perform a Bayesian NMA to evaluate the relative efficacy and safety of ABA, TER, OBP, IBP, DEN, and PLA/CTRL in postmenopausal women with osteoporosis, specifically for lumbar spine, femoral neck, and total hip BMD improvement, as well as adverse event incidence. The findings are intended to support clinical decision-making, risk assessment, and individualized treatment strategies, and to provide evidence for future research in female osteoporosis interventions.

## 2 Materials and methods

### 2.1 Literature search strategy

This systematic review and NMA was conducted following the PRISMA 2020 guidelines and Cochrane Collaboration recommendations to ensure transparency and methodological rigor (16). The study protocol was registered in PROSPERO (CRD420261398700).

Electronic searches were performed in PubMed, Web of Science, and the Cochrane Library from database inception through May 2026. The search strategy combined MeSH terms and free-text keywords covering postmenopausal osteoporosis and interventions (ABA, TER, OBP, IBP, DEN, etc.). To ensure completeness, references of published systematic reviews, meta-analyses, and relevant studies were manually screened.

### 2.2 Inclusion and exclusion criteria

#### 2.2.1 Inclusion criteria

1. Study design: randomized controlled trials (RCTs);
2. Population: postmenopausal women with osteoporosis;
3. Interventions: ABA, TER, OBP, IBP, DEN; comparator: PLA/CTRL;
4. Outcomes: changes in lumbar spine, femoral neck, and total hip BMD (%) measured by dual-energy X-ray absorptiometry or equivalent methods (17–20); safety outcomes including all adverse events (AEs) and serious adverse events (SAEs) (21, 22);
5. Follow-up: outcome data reported at approximately 12 months, or at the closest comparable time point when 12-month data were unavailable. Studies reporting only substantially longer-term outcomes without extractable data near the target time point were excluded to reduce time-point heterogeneity.

#### 2.2.2 Exclusion criteria

1. Non-RCTs (observational studies, retrospective analyses, etc.);
2. Studies including men or mixed-sex populations without separate female data;
3. Studies with incomplete or non-extractable outcomes;
4. Duplicate publications or overlapping datasets.

### 2.3 Data extraction and quality assessment

Two independent reviewers (XX, XX) extracted data using a pre-designed form, including author, year, sample size, patient characteristics, interventions, follow-up duration, and primary outcomes. Discrepancies were resolved by a third reviewer (XX).

Efficacy was assessed by BMD changes (%), and safety by all reported AEs and SAEs. Missing or incomplete data were handled according to the Cochrane Handbook (Section 6.5.2) with appropriate variance conversions or estimations. When mean differences and P values were reported, standard errors were calculated using formulas from Section 6.5.2.3; if variance was not reported, a conservative SD of 30 was assumed (23).

Risk of bias was assessed using the Cochrane RoB 2.0 tool, evaluating randomization, allocation concealment, blinding, incomplete outcome data, selective reporting, and other biases. Each domain was rated as low, some concerns, or high risk (20, 24).

### 2.4 Statistical analysis

Pairwise meta-analyses were performed in Stata 18.0, calculating standardized mean differences (SMDs) or odds ratios (ORs) with 95% confidence intervals (CIs). Heterogeneity was assessed using the I² statistic; I² <50% indicated low heterogeneity (fixed-effect model), I² ≥50% indicated high heterogeneity (random-effects model).

Bayesian NMA was conducted using R 4.3.1 with the gemtc and BUGSnet packages (25–27). Network diagrams illustrated direct and indirect comparisons. Relative efficacy was evaluated using a consistency model, and cumulative ranking probabilities (SUCRA) were calculated. Node-splitting assessed consistency between direct and indirect evidence, and the deviance information criterion (DIC) was used to compare model fit. The potential impact of methodological quality was assessed through risk-of-bias evaluation and was considered in the interpretation of the findings (27).

## 3 Results

**Figure 1:**
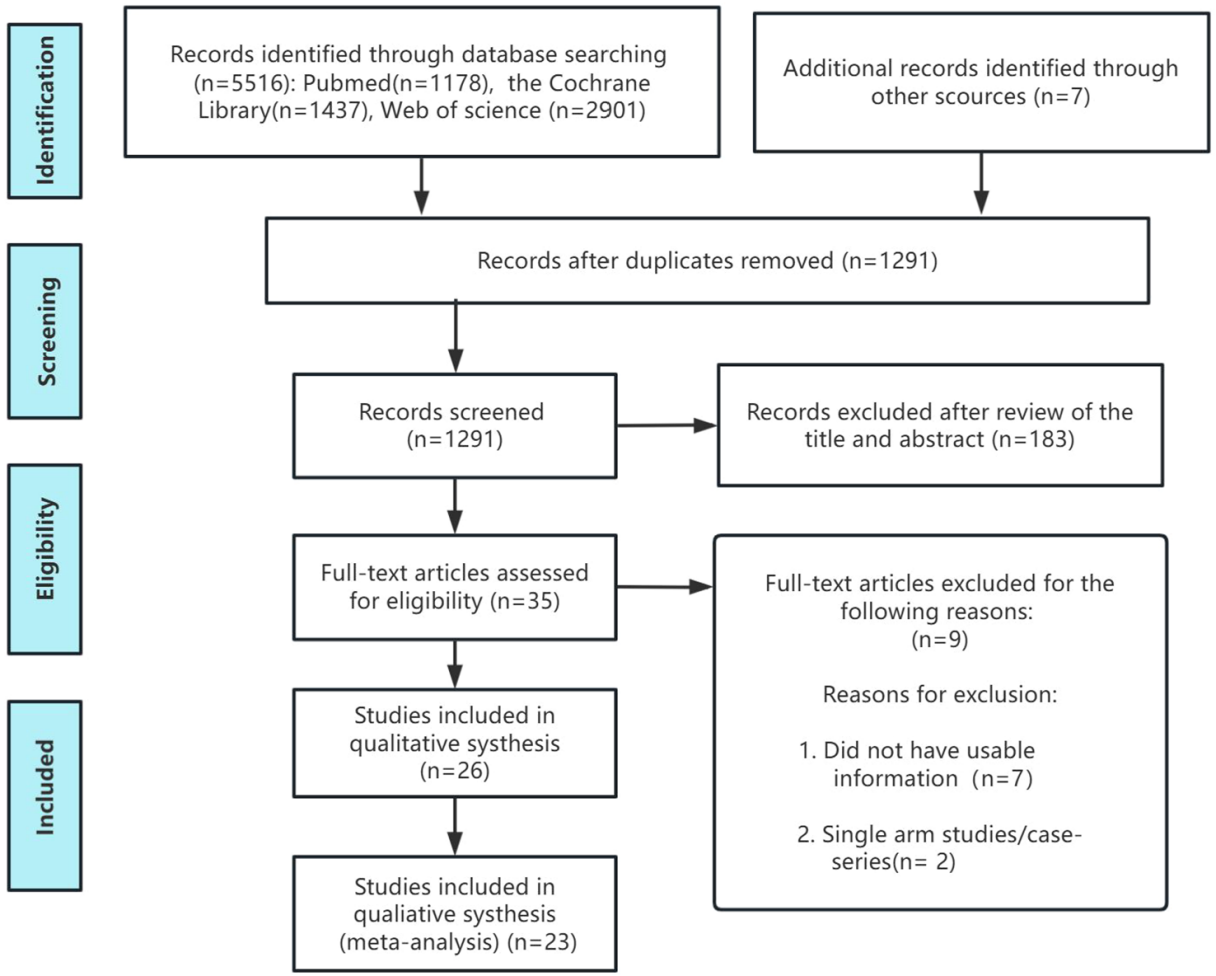
Flow diagram of the study selection process for the network meta-analysis.

**Figure 1** illustrates the flow diagram for the selection of randomized controlled trials (RCTs) included in this study. During the literature search, a total of 5, 516 records were identified. After removing duplicates, 1, 291 unique studies remained for further assessment. Following full-text review and application of pre-specified inclusion criteria, several studies were excluded due to low relevance to the research question. For example, Dennis M. Black 2007 (28) and Richard M. Jacques 2012 (29) reported results from the same cohort at different follow-up times; thus, the former was retained and the latter excluded. S. R. Cummings 1998 (30) was excluded due to an early study period (before 2000). Jonathan D. Adach 2009 (31) focused primarily on upper gastrointestinal tolerability; S. Adami 2008 (32) involved raloxifene treatment; Douglas C. Bauer 2006 (33) reported fracture incidence as the primary endpoint; John P. Bilezikian 2019 (34) included patients with mild-to-moderate renal impairment receiving abaloparatide; S. Boonen 2011 (35) emphasized age-related variations; P. D. Delmas 2004 (36) only reported three-year outcomes. Additionally, N. Freemantle 2012 (37) involved combined denosumab and alendronate therapy; S. T. Harris 1999 (38) was an early study; David L. Kendler 2010 (39) and Bente L. Langdahl 2017 (40) focused on alendronate treatment for ≥6 months; Michael R. McClung 2012 (41) did not report extractable BMD and safety outcomes at approximately 12 months or the closest comparable time point required for the present analysis; inclusion of longer-term outcome data would have introduced additional time-point heterogeneity.; P. D. Miller 2016 (42) examined conversion from oral bisphosphonates to denosumab or zoledronic acid; C. Roux 2014 (43) included postmenopausal women with poor adherence to alendronate; Huan Wang 2025 (44) evaluated teriparatide dosing and timing optimization; Ian R. Reid 2024 (45) had an excessively long baseline follow-up (6–10 years); Ramchand S. K. 2026 (46) studied combination therapy of PTH receptor agonist (teriparatide 20 µg) and RANKL inhibitor (denosumab 60 mg). All of these studies were excluded from the final analysis.

### 3.1 Study characteristics and study quality

**Figure 2:**
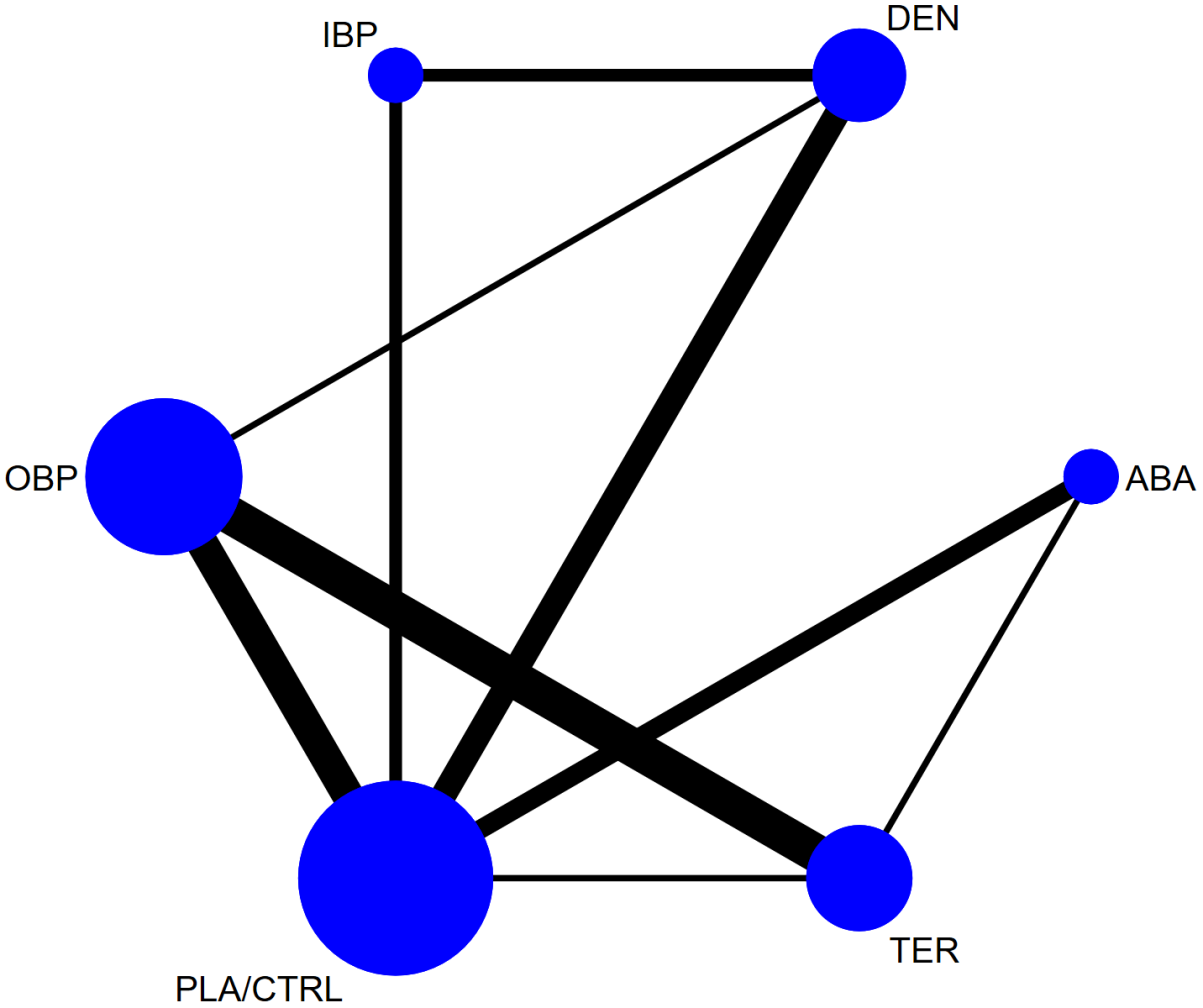
Network plot of the included trials.

**Figure 2** presents the network diagram of the included studies, where each osteoporosis intervention was compared with at least one placebo and with at least one active drug within the network. Node-splitting analysis indicated no significant inconsistency (P > 0.05). **Supplementary Table 1** shows statistical analyses for consistency and inconsistency models, demonstrating good agreement. The primary endpoints (lumbar spine, femoral neck, and total hip BMD) were mostly assessed at 12 months, with a few studies reporting longer or shorter follow-up. For comparability, the data closest to 12 months were pooled. **Supplementary Figure 1** shows forest plots for all outcomes; **Supplementary Figure 2** presents the network graph, where node size represents the number of participants and edge width reflects the number of direct comparisons. **Supplementary Figure 3** depicts funnel plots for bias assessment, which showed no apparent publication bias upon visual inspection.

**Table 1** summarizes the characteristics of the 23 RCTs included in the NMA (28, 47–68). Studies were published between 2002 and 2026, with postmenopausal women ranging from 80 to 7, 808 participants and follow-up periods of 1–3 years. Most trials compared interventions with placebo and/or calcium and vitamin D. Direct head-to-head comparisons among the five treatment strategies were limited. Except for multicenter studies, most trials were conducted in the United States. Doses reflected standard clinical regimens: risedronate (oral, 35 mg/week or 5 mg/day), alendronate (70 mg/week or 10 mg/day), teriparatide (20 µg/day), denosumab (60 mg every 6 months), abaloparatide (80 µg/day), and zoledronic acid (5 mg/year). These regimens were widely applied in the included studies, forming the basis for comparative analyses.

**Table 1:** The Main Features of the Articles.

Author, year	Study design	Country	Treatment	Comparator	Background treatment	Group/Patient	Length of interv
Kendler 2018 (51)	RCT	Multi-country	Teriparatide 20ug, subcutaneous daily injection	Risedronate 35 mg, weekly oral administration	Calcium + Vitamin D	G1: 680 G2: 680	24m
Sierra 2017 (52)	RCT	Multi-country	Teriparatide 20ug, subcutaneous daily injection	Risedronate 35 mg, weekly oral administration	Calcium + Vitamin D	G1: 680 G2: 680	36m
Black 2007 (28)	RCT	Multi-country	Zoledronic Acid 5 mg, yearly intravenous injection	Placebo	Calcium + Vitamin D	G1: 3875 G2: 3861	18m
Anastasilakis 2015 (55)	RCT	USA	Denosumab 60 mg, sub cutaneous injection every 6 months (q6m)	Zoledronic Acid 5 mg, yearly intravenous injection	Calcium + Vitamin D	G1:34 G2: 30	24m
Evans 2003 (67)	RCT	Multi-country	Alendronate 10 mg, oral daily administration	Placebo	Calcium + Vitamin D	G1: 95 G2: 49	24m
Austin 2012 (58)	RCT	Multi-country	Denosumab 60 mg, sub cutaneous injection every 6 months (q6m)	Placebo	Calcium + Vitamin D	G1: 3902 G2: 3906	24m
Body 2002 (68)	RCT	Belgium	Teriparatide 20ug, subcutaneous daily injection	Alendronate 10 mg, oral daily administration	Calcium + Vitamin D	G1: 73 G2: 73	36m
Bone 2008 (62)	RCT	Multi-country	Denosumab 60 mg, sub cutaneous injection every 6 months (q6m)	Placebo	Calcium + Vitamin D	G1: 166 G2: 166	12m
Brown 2009 (61)	RCT	Multi-country	Ibandronate 150 mg, oral monthly administration	Alendronate 10 mg, oral daily administration	Calcium + Vitamin D	G1: 594 G2: 595	12m
Chesnut 2004 (66)	RCT	Multi-country	Alendronate 10 mg, oral daily administration	Placebo	Calcium + Vitamin D	G1: 975 G2: 977	36m
Cummings et al., 2009 (60)	RCT	Multi-country	Denosumab 60 mg, sub cutaneous injection every 6 months (q6m)	Placebo	Calcium + Vitamin D	G1: 3902 G2: 3906	14m
Greenspan 2015 (54)	RCT	Pittsburgh	Zoledronic Acid 5 mg, yearly intravenous injection	Placebo	Calcium + Vitamin D	G1: 89 G2: 92	24m
Hadji 2012 (56)	RCT	Multi-country	Teriparatide 20ug, subcutaneous daily injection	Risedronate oral 35 mg, weekly oral administration	Calcium + Vitamin D	G1:259 G2: 269	24m
McClung 2005 (65)	RCT	USA	Teriparatide 20ug, subcutaneous daily injection	Risedronate oral 35 mg, weekly oral administration	Calcium + Vitamin D	G1: 102 G2: 101	24m
McClung 2018 (50)	RCT	Multi-country	Abaloparatide 80 µg, daily subcutaneous injection	Placebo	Calcium + Vitamin D	G1: 824 G2: 821	24m
McClung 2006 (64)	RCT	Multi-country	Denosumab 60 mg, sub cutaneous injection every 6 months (q6m)	Placebo	Calcium + Vitamin D	G1: 47 G2: 46	36m
Miller 2016 (42)	RCT	Multi-country	Abaloparatide 80 µg, daily subcutaneous injection	Placebo	Calcium + Vitamin D	G1: 822 G2: 818	24m
Välimäki 2007 (63)	RCT	Multi-country	Alendronate 10 mg, oral daily administration	Placebo	Calcium + Vitamin D	G1: 114 G2: 56	24m
Bock 2012 (57)	RCT	Germany	Ibandronate 150 mg, oral monthly administration	Placebo	Calcium + Vitamin D	G1: 35 G2: 33	18m
Saag 2020 (49)	RCT	USA	Abaloparatide 80 µg, daily subcutaneous injection	1 Teriparatide 20ug, subcutaneous daily injection 2 Placebo	Calcium + Vitamin D	G1: 94 G2: 99 G3: 103	24m
Clung 2009 (59)	RCT	USA	Ibandronate 150 mg, oral monthly administration	Placebo	Calcium + Vitamin D	G1: 77 G2: 83	24m
Chiba 2022 (48)	RCT	Japan	Teriparatide 20ug, subcutaneous daily injection	Alendronate 10 mg, oral daily administration	Calcium + Vitamin D	G1: 46 G2: 40	36m
Tang 2026 (47)	RCT	China	Zoledronic Acid 5 mg, yearly intravenous injection	Denosumab 60 mg, sub cutaneous injection every 6 months (q6m)	Calcium + Vitamin D	G1: 40 G2: 40	24m

**Figure 3:**
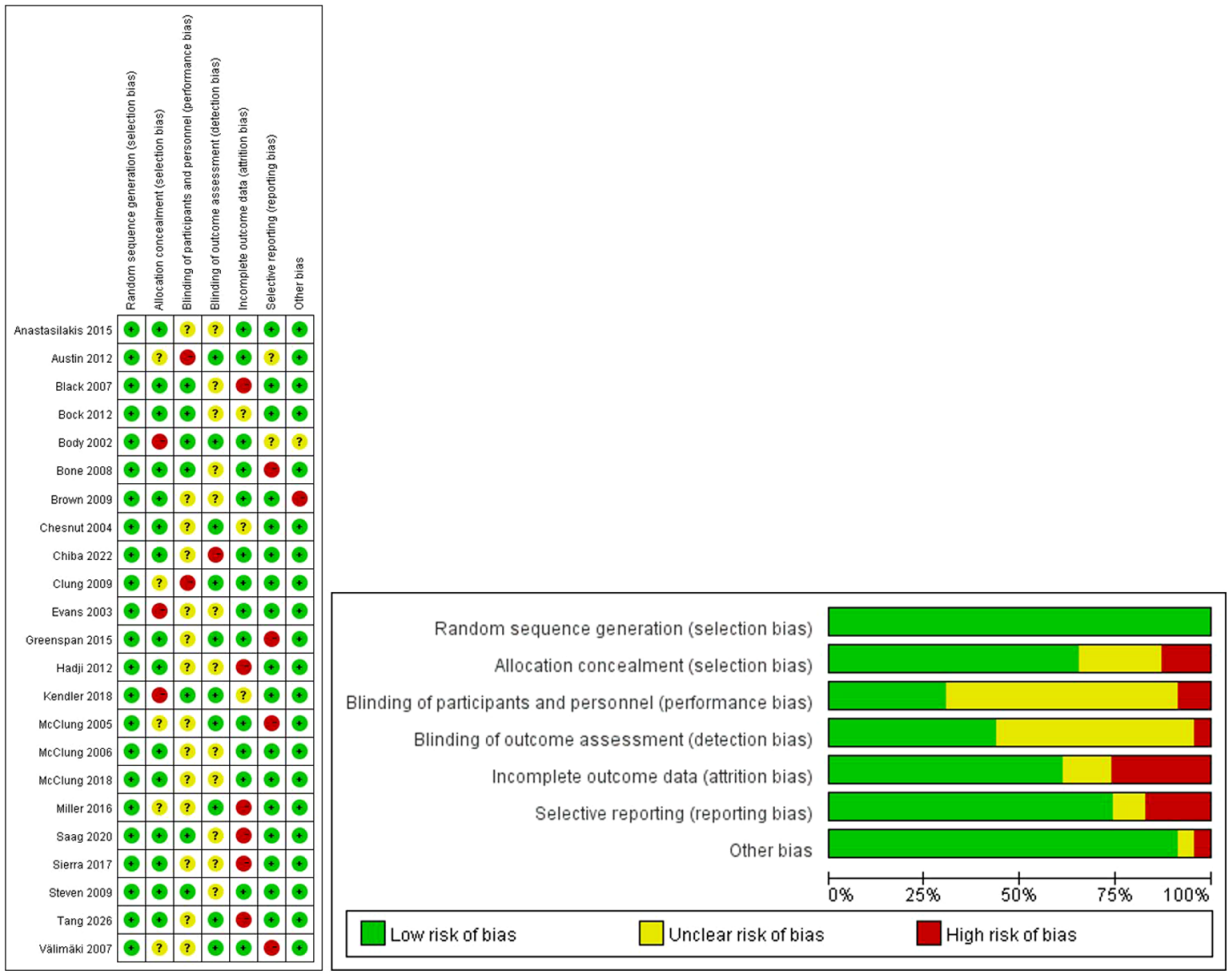
Risk-of-bias summary for the included randomized controlled trials: reviewers’ judgments for each risk-of-bias domain across studies.XXX.

**Figure 3** presents risk of bias assessment based on the Cochrane Collaboration guidelines. Domains assessed included random sequence generation, allocation concealment, and completeness of outcome reporting. Overall, most studies inadequately reported randomization and allocation concealment details. Some studies did not clearly report all pre-specified outcomes, which is critical for assessing reporting bias. Due to limited methodological transparency, it was not possible to definitively classify these studies as low or high risk of bias. Consequently, most studies were rated as “unclear risk” for selection and reporting biases.

### 3.2 Lumbar spine BMD

**Figure 4:**
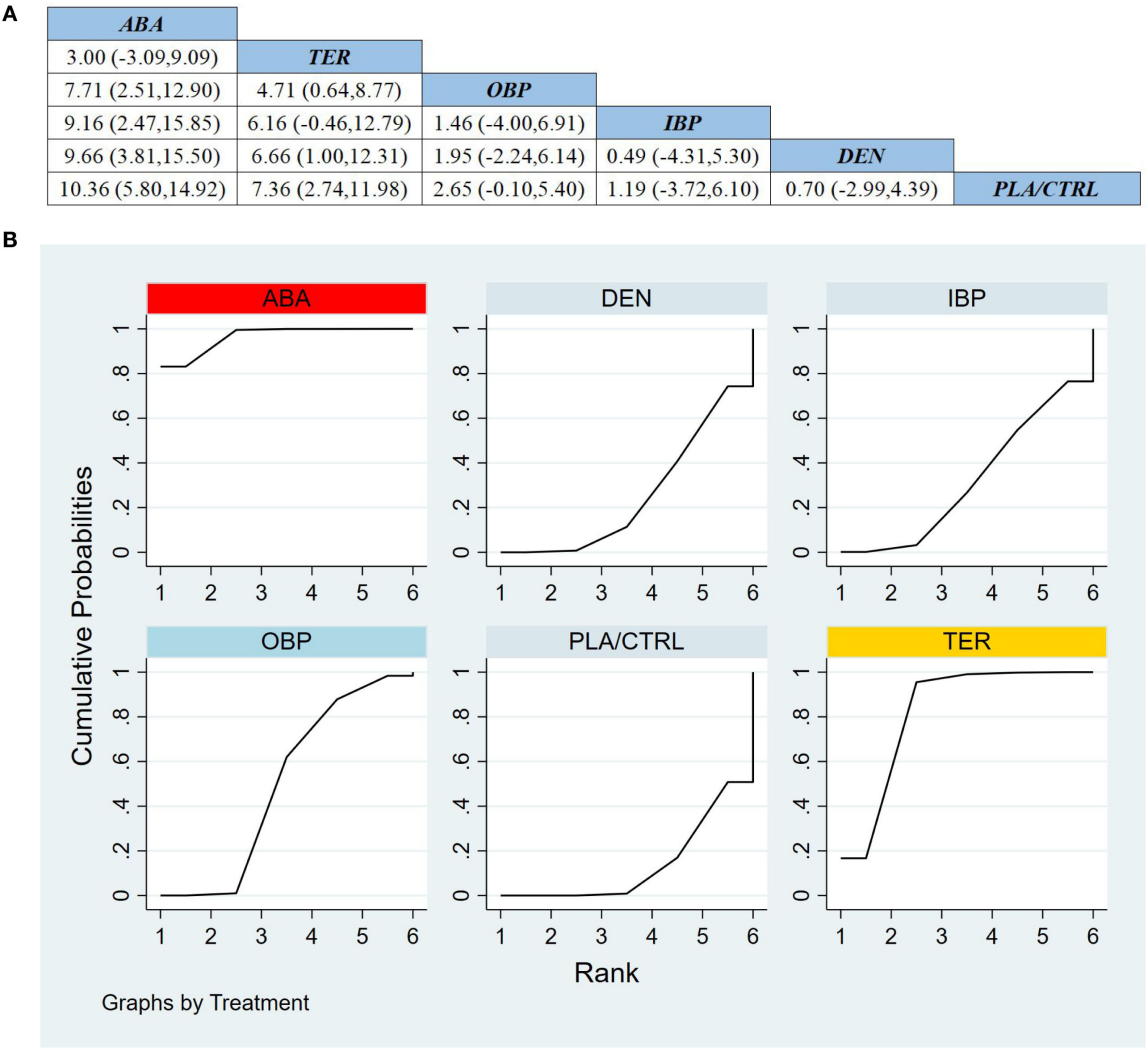
**(a)** League table for lumbar spine BMD. **(b)** SUCRA ranking probabilities for percentage change in lumbar spine BMD.

In this network meta-analysis, six interventions were included: abaloparatide (ABA), teriparatide (TER), oral bisphosphonates (OBP), intravenous bisphosphonates (IBP), denosumab (DEN), and placebo/conventional therapy (PLA/CTRL). League table (**Figure 4a**) results indicated that, compared with PLA/CTRL, ABA increased lumbar spine BMD by 10.36% (95% CI 5.80–14.92%), TER by 7.36% (95% CI 2.74–11.98%), OBP by 2.65% (95% CI –0.10–5.40%), IBP by 1.19% (95% CI –3.72–6.10%), and DEN by 0.70% (95% CI –2.99–4.39%).

To further quantify the relative efficacy of the interventions on lumbar spine BMD, cumulative ranking curves (SUCRA) were generated. **Figure 4b** shows the lumbar spine BMD results, where red indicates the highest rank, yellow the second, and blue the third. The ranking results reveal significant differences in treatment efficacy: higher rankings correspond to better effects. The SUCRA curves indicated that ABA had a cumulative probability approaching 1, suggesting the most favorable effect on lumbar spine BMD, followed by TER; OBP, IBP, and DEN showed intermediate effects, and PLA/CTRL ranked lowest. These findings indicate that ABA and TER have significant advantages in improving lumbar spine BMD in postmenopausal women, whereas OBP, IBP, and DEN have comparatively limited effects.

### 3.3 Femoral neck BMD

**Figure 5:**
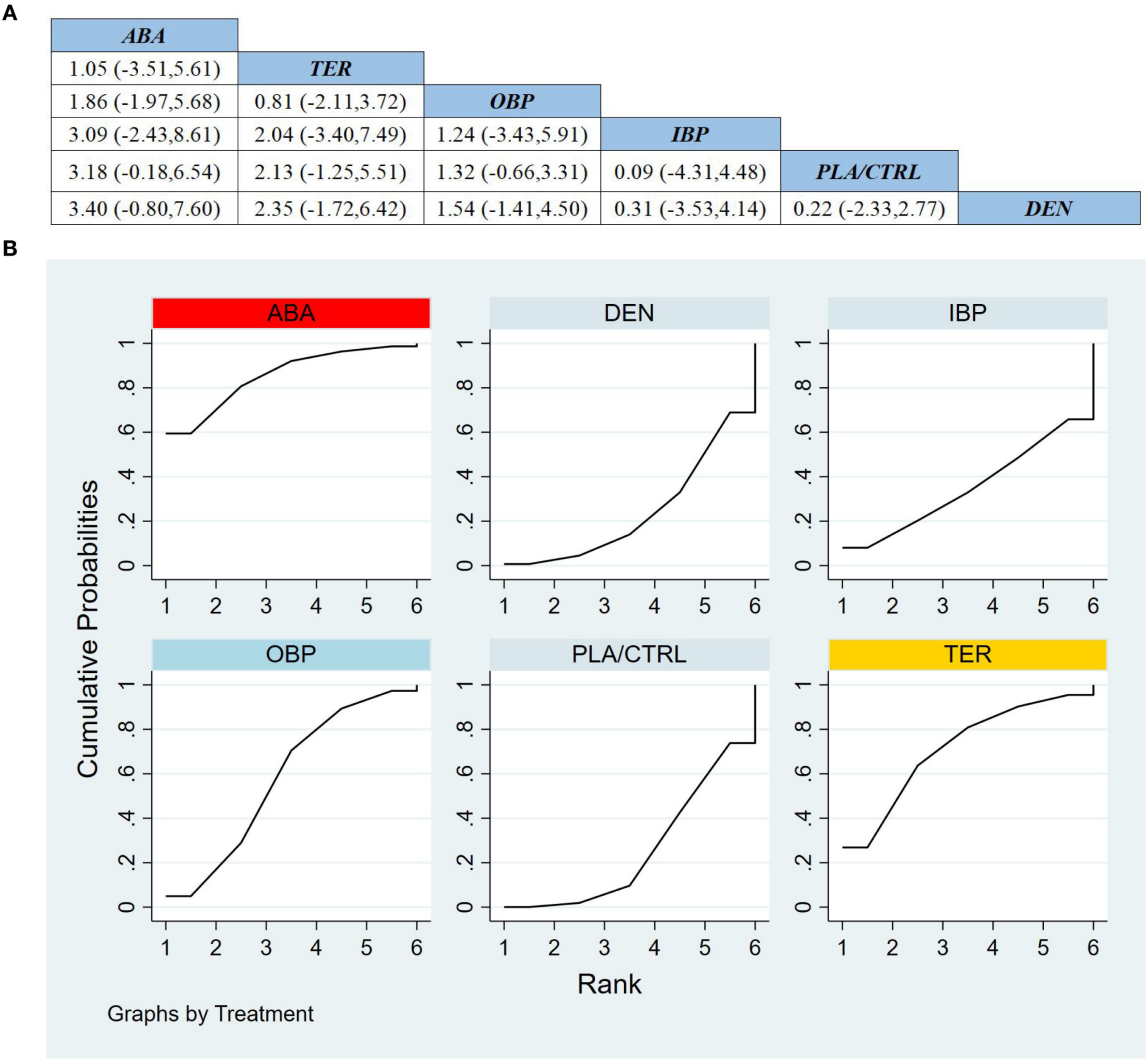
**(a)** League table for femoral neck BMD. **(b)** SUCRA ranking probabilities for percentage change in femoral neck BMD.

The same six interventions were evaluated for femoral neck BMD. League table (**Figure 5a**) results showed that, compared with PLA/CTRL, ABA increased femoral neck BMD by 3.40% (95% CI –0.80–7.60%), TER by 2.35% (95% CI –1.72–6.42%), OBP by 1.54% (95% CI –1.41–4.50%), IBP by 1.32% (95% CI –0.66–3.31%), PLA/CTRL vs DEN by 0.31% (95% CI –3.53–4.14%), and DEN by 0.22% (95% CI –2.33–2.77%).

SUCRA (**Figure 5b**) were applied to further assess relative efficacy. ABA had the highest cumulative probability, indicating the most favorable effect on femoral neck BMD, followed by TER; OBP, IBP, and PLA/CTRL showed intermediate effects, while DEN ranked lowest. This demonstrates that ABA and TER have significant advantages for improving femoral neck BMD, whereas other interventions provide relatively limited benefit.

### 3.4 Total hip BMD

**Figure 6:**
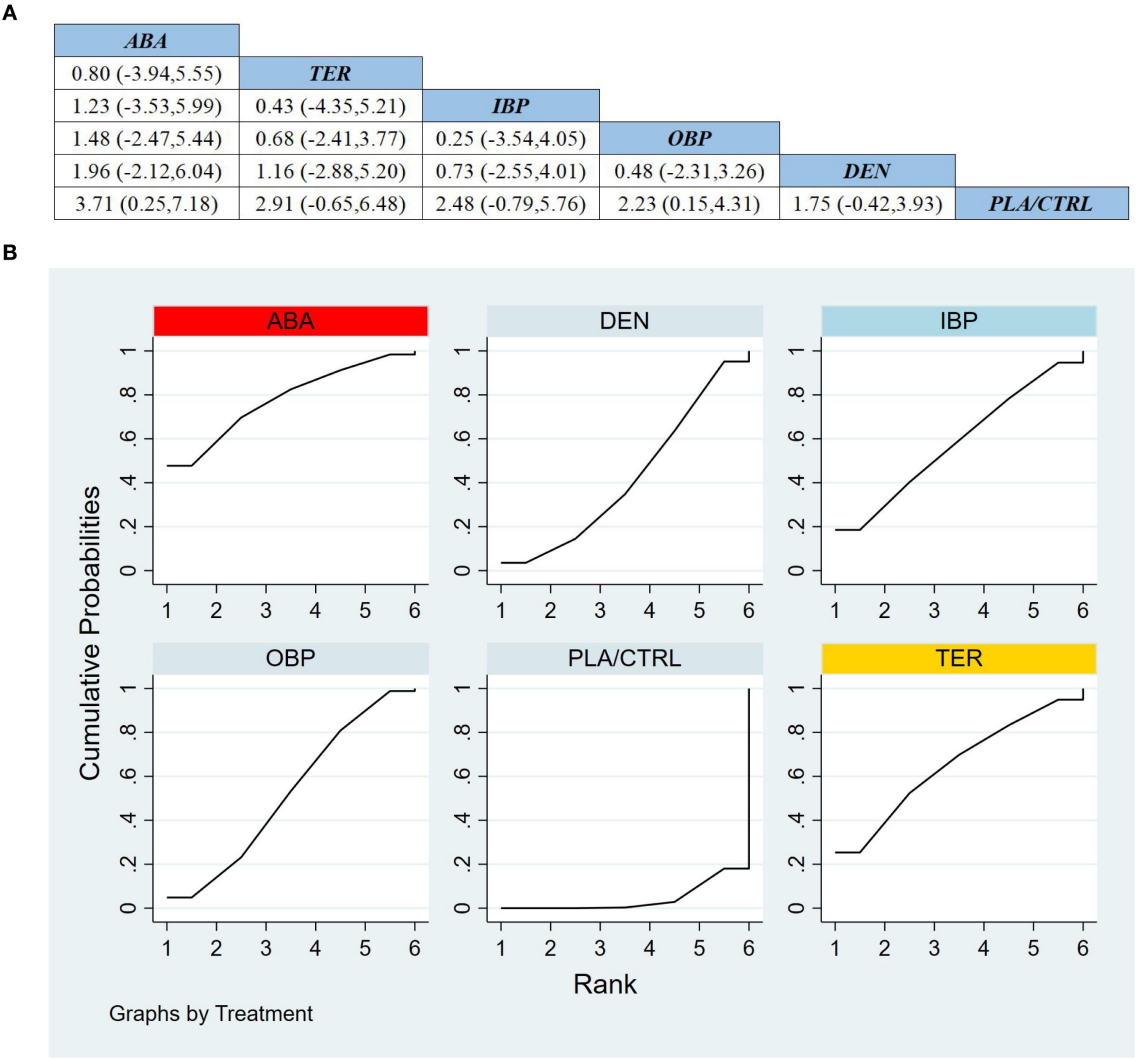
**(a)** League table for total hip BMD. **(b)** SUCRA ranking probabilities for percentage change in total hip BMD.

The same six interventions were assessed for total hip BMD. League table results (**Figure 6a**) indicated that, compared with PLA/CTRL, ABA increased total hip BMD by 3.71% (95% CI 0.25–7.18%), TER by 2.91% (95% CI –0.65–6.48%), IBP by 2.48% (95% CI –0.79–5.76%), OBP by 2.23% (95% CI 0.15–4.31%), and DEN by 1.75% (95% CI –0.42–3.93%).

SUCRA rankings were generated to further summarize the relative ranking probabilities for total hip BMD improvement. **Figure 6b** presents the cumulative ranking probabilities for total hip BMD. ABA showed the highest cumulative ranking probability, followed by TER and IBP, suggesting that these interventions had a higher probability of ranking favorably for total hip BMD improvement within the treatment network. However, SUCRA should be interpreted together with the corresponding effect estimates and confidence intervals, because it reflects ranking probability rather than the absolute magnitude of treatment effect. Therefore, although ABA ranked highest in the SUCRA analysis, the total hip BMD findings should be interpreted cautiously, particularly because several comparisons had wide confidence intervals.

### 3.5 All adverse events

**Figure 7:**
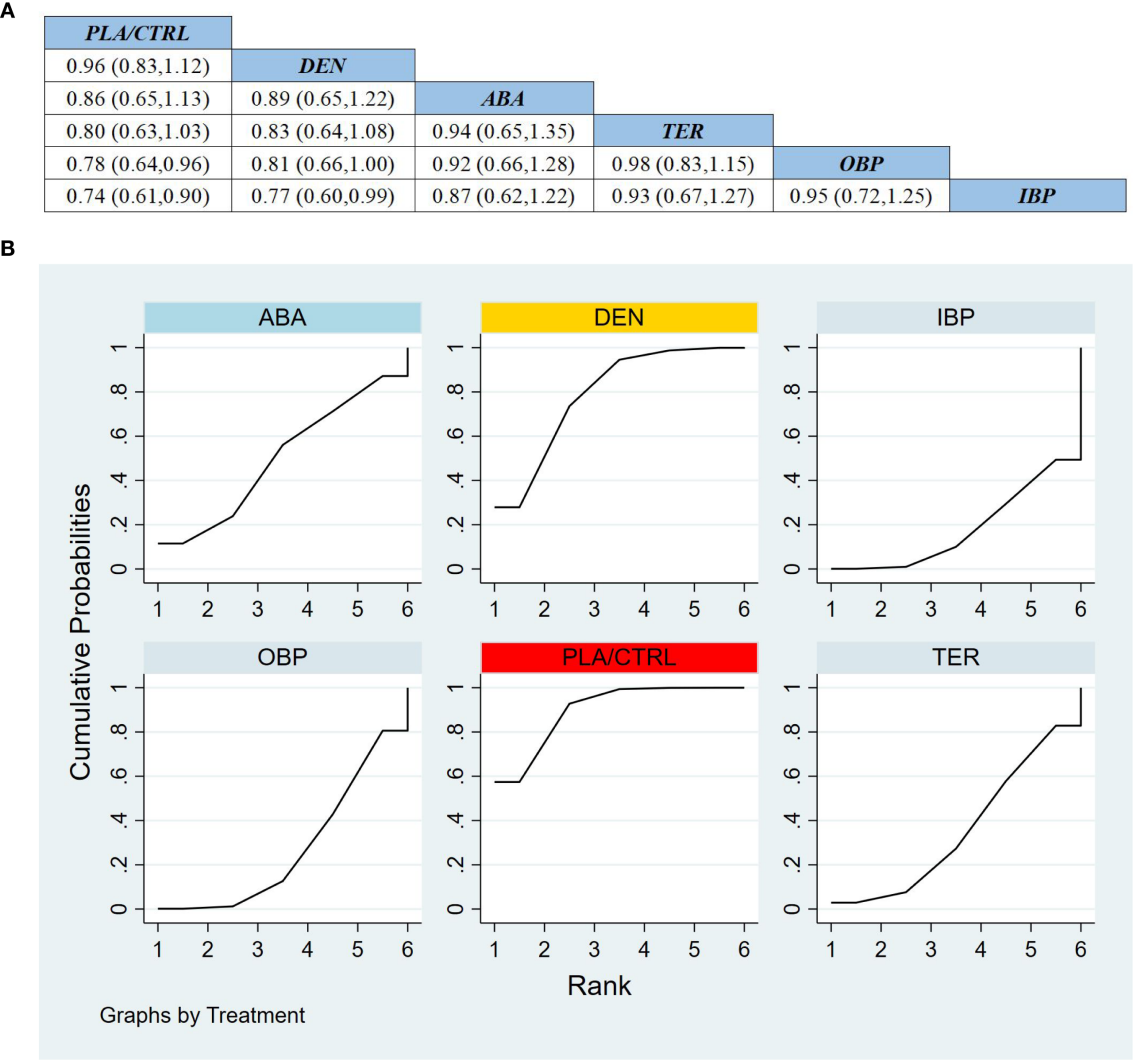
**(a)** League table for all adverse events. **(b)** SUCRA ranking probabilities for all adverse events.

The relative safety of six interventions for all adverse events was evaluated. League table (**Figure 7a**) data were as follows: PLA/CTRL vs IBP, RR 0.74 (95% CI 0.61–0.90); DEN vs IBP, RR 0.77 (95% CI 0.60–0.99); ABA vs IBP, RR 0.87 (95% CI 0.62–1.22); TER vs IBP, RR 0.92 (95% CI 0.66–1.28); OBP vs IBP, RR 0.95 (95% CI 0.72–1.25).

To further quantify the relative safety of the interventions regarding all AEs, SUCRA were applied. The SUCRA (**Figure 7b**) curves indicated that TER and OBP had the highest cumulative probabilities, suggesting that these two drugs were the safest in reducing all AEs, followed by ABA, DEN, and PLA/CTRL, with IBP having the highest risk.

### 3.6 Serious adverse events

**Figure 8:**
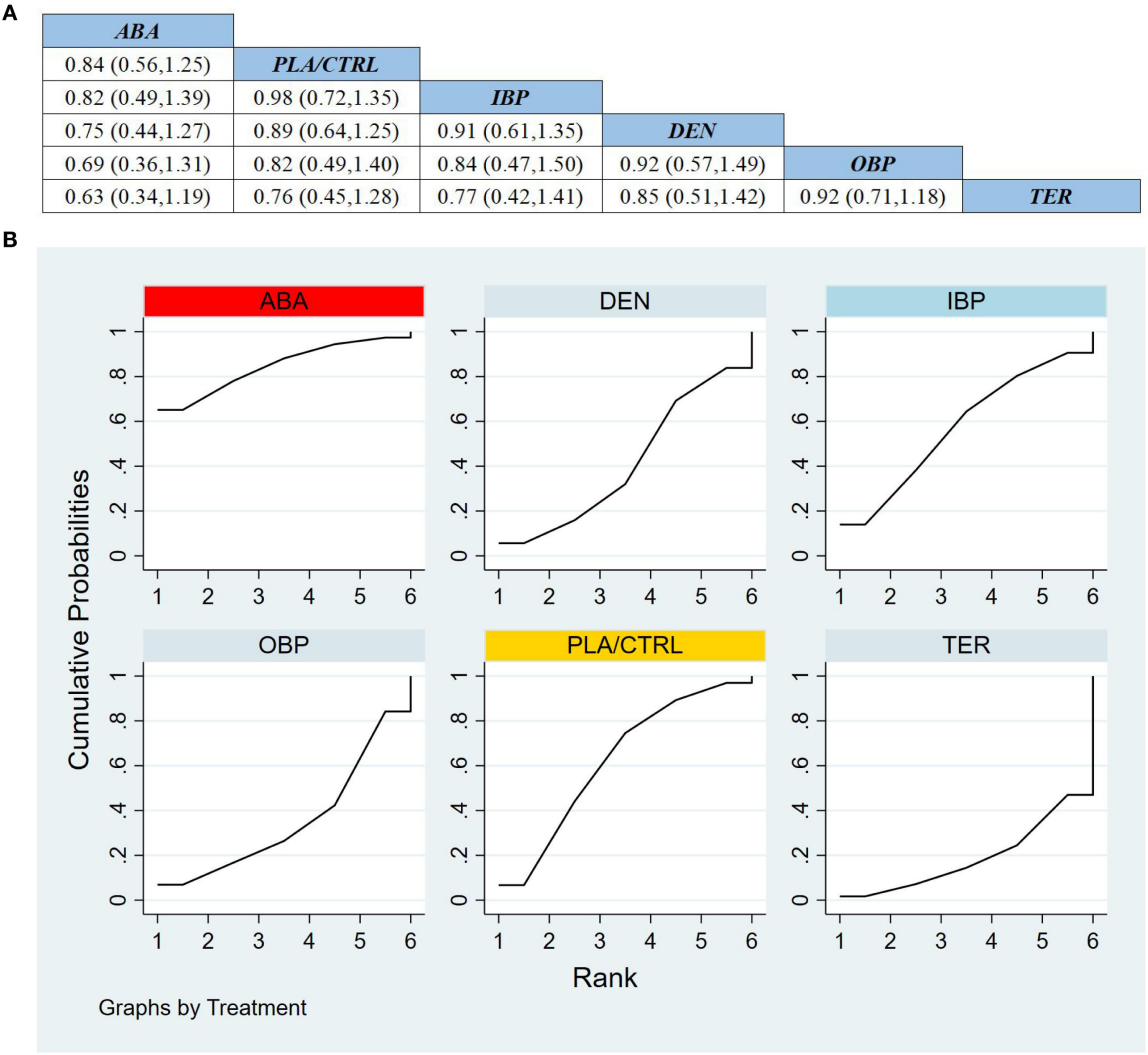
**(a)** League table for serious adverse events. **(b)** SUCRA ranking probabilities for serious adverse events.

The relative safety of the six interventions for serious adverse events was evaluated. League table (**Figure 8a**) data were: ABA vs TER, RR 0.63 (95% CI 0.34–1.19); ABA vs OBP, RR 0.69 (95% CI 0.36–1.31); ABA vs DEN, RR 0.75 (95% CI 0.44–1.27); ABA vs IBP, RR 0.82 (95% CI 0.49–1.39); ABA vs PLA/CTRL, RR 0.84 (95% CI 0.56–1.25).

SUCRA were used to further quantify relative safety for SAEs. The SUCRA (**Figure 8b**) curves showed that ABA and PLA/CTRL had the highest cumulative probabilities, indicating the lowest risk of serious adverse events, followed by TER and OBP; IBP and DEN were associated with higher risk.

## 4 Discussion

This network meta-analysis systematically evaluated the relative efficacy and safety of abaloparatide (ABA), teriparatide (TER), oral bisphosphonates (OBP), intravenous bisphosphonates (IBP), denosumab (DEN), and placebo/conventional therapy (PLA/CTRL) in postmenopausal women with osteoporosis, focusing on improvements in lumbar spine, femoral neck, and total hip bone mineral density (BMD). By integrating direct and indirect comparisons, this study provides an updated comparative ranking of multiple interventions in a female osteoporosis population, offering evidence to guide individualized clinical drug selection.

Results indicated that ABA was most effective in improving lumbar spine and femoral neck BMD, followed by TER, highlighting the pronounced effect of anabolic agents on bone mass at the spine and femoral neck. This finding aligns with the mechanism of ABA and TER, which stimulate bone formation rather than merely inhibiting bone resorption, rapidly improving bone microarchitecture and enhancing bone strength. For total hip BMD, ABA showed the highest ranking probability, while OBP also demonstrated a statistically significant improvement compared with PLA/CTRL. These findings suggest that both anabolic and antiresorptive agents may contribute to total hip BMD improvement, but the relative ranking should be interpreted together with the corresponding effect estimates and confidence intervals. IBP and DEN showed relatively modest improvements across most BMD endpoints but were still superior to controls. DEN demonstrated intermediate efficacy for total hip BMD, potentially related to its mechanism and dosing interval, while the variability in IBP effects may be attributable to individual adherence and plasma drug fluctuations. Overall, anabolic agents had notable effects on the spine and femoral neck, while antiresorptive agents were advantageous for total hip BMD, indicating that clinical treatment should be individualized based on high-risk fracture sites and patient-specific risk factors. The clinical relevance of these findings should be interpreted with caution. Although BMD is an important surrogate marker and is widely used in osteoporosis trials, fracture prevention remains the primary goal of osteoporosis treatment. Improvements in BMD at the lumbar spine, femoral neck, or total hip do not necessarily correspond to equivalent reductions in vertebral, nonvertebral, or hip fracture risk. This is particularly important because different agents may affect bone quality, microarchitecture, cortical and trabecular compartments, and bone turnover through distinct mechanisms beyond changes in areal BMD. Therefore, the treatment rankings in the present network meta-analysis should be understood primarily as rankings for site-specific BMD improvement and safety outcomes, rather than as direct evidence of comparative fracture-prevention efficacy. Future network meta-analyses incorporating fracture outcomes are needed to determine whether the observed BMD advantages translate into clinically meaningful fracture-risk reduction.

Regarding safety, TER and OBP showed relatively favorable ranking probabilities for all adverse events, while ABA and PLA/CTRL showed relatively favorable ranking probabilities for serious adverse events. However, these findings should be interpreted cautiously. Several comparisons for AEs and SAEs had wide confidence intervals, indicating uncertainty in the relative safety estimates. In addition, safety outcomes were not always reported with the same level of detail across trials, and the included studies may have differed in follow-up duration, adverse-event definitions, and patient risk profiles. Therefore, the safety results should be regarded as exploratory comparative evidence rather than definitive proof that one intervention is safer than another. Clinical treatment decisions should continue to consider known drug-specific safety concerns, patient comorbidities, renal function, adherence, and prior adverse-event history.

Taken together, these results suggest that ABA and TER have favorable efficacy in improving lumbar spine and femoral neck BMD in postmenopausal women with osteoporosis. For total hip BMD, ABA showed the highest ranking probability, while OBP also demonstrated a statistically significant improvement compared with PLA/CTRL. However, these findings should be interpreted primarily as evidence of site-specific BMD improvement rather than direct evidence of fracture-risk reduction. Regarding safety, TER and OBP showed relatively favorable ranking probabilities for all AEs, and ABA showed a relatively favorable ranking probability for SAEs. Nevertheless, because several safety comparisons had wide confidence intervals, these results should be interpreted cautiously. Treatment decisions should consider individual fracture risk, high-risk skeletal sites, drug adherence, comorbidities, contraindications, and prior adverse event history.

Previous NMAs have primarily focused on male or mixed-sex populations, with limited female-specific data (15, 69). By integrating RCT data in postmenopausal women, this study provides sex-specific evidence and comprehensively evaluates lumbar spine, femoral neck, and total hip BMD. Consistent with prior research, anabolic agents demonstrated superior efficacy at the spine and femoral neck compared to traditional antiresorptive drugs, while antiresorptive agents maintained advantages in hip bone mass, supporting the relationship between drug mechanism and site-specific bone structure. The present findings are consistent with recent evidence supporting the clinical value of anabolic therapy in osteoporosis. Bonifacio et al. (11) reported that abaloparatide reduced vertebral fracture risk and substantially increased lumbar spine BMD, although gains at the hip and femoral neck were smaller and more variable. In agreement with this evidence, our network meta-analysis showed that abaloparatide ranked highest for lumbar spine and femoral neck BMD improvement among the included interventions. Cipolloni et al. (12) further emphasized the anabolic-first strategy for patients at very high fracture risk, suggesting that early use of anabolic agents may result in faster and more pronounced fracture-risk reduction. Our results support this concept from the perspective of BMD improvement, as both abaloparatide and teriparatide showed favorable efficacy at the lumbar spine and femoral neck. Nevertheless, our study extends these previous reviews by simultaneously comparing multiple commonly used pharmacological strategies, including abaloparatide, teriparatide, denosumab, oral bisphosphonates, intravenous bisphosphonates, and placebo/conventional therapy, and by demonstrating site-specific differences in treatment ranking. Notably, oral bisphosphonates ranked highest for total hip BMD, suggesting that antiresorptive therapy may still play an important role in patients with predominant hip-related fracture risk.

Potential sources of heterogeneity across the included trials should also be considered when interpreting the present network meta-analysis. Although all included studies enrolled postmenopausal women with osteoporosis, important clinical characteristics varied across trials, including baseline BMD, age, baseline fracture risk, years since menopause, and previous osteoporosis treatment. These factors may act as effect modifiers and influence the magnitude of BMD response to anabolic or antiresorptive therapy. For example, patients with lower baseline BMD or higher fracture risk may show different absolute BMD gains compared with patients with milder disease, while prior bisphosphonate exposure may attenuate or modify the subsequent response to anabolic agents or denosumab. Such differences may affect the transitivity assumption underlying indirect comparisons in network meta-analysis. Although node-splitting analysis did not detect significant inconsistency, the possibility of residual clinical heterogeneity cannot be excluded. Subgroup analyses or meta-regression based on these variables were considered; however, they were not feasible because these characteristics were inconsistently reported across studies and because several treatment nodes included only a limited number of trials. Therefore, the indirect comparisons and treatment rankings should be interpreted with appropriate caution.

However, this study has several limitations: (1) some therapeutic agents lack direct head-to-head comparisons, which may affect the rigor of the results and the reliability of the conclusions; (2) although follow-up durations in the included studies were generally close to 12 months, some variability remained, potentially impacting the assessment of long-term effects; (3) the included studies spanned a long time period, and some reported insufficient methodological details, which may lead to differences in study design, patient characteristics, and data collection methods, thereby affecting the quality of the results; (4) oral bisphosphonates and intravenous bisphosphonates were analyzed as two broad treatment categories according to route of administration and shared antiresorptive mechanism. This classification was adopted to maintain network connectivity and to allow stable comparative estimates, because the available evidence for individual bisphosphonate agents was sparse and unevenly distributed across outcomes. However, individual bisphosphonates differ in potency, pharmacokinetic characteristics, dosing regimens, adherence requirements, and clinical efficacy. Therefore, combining these agents may have introduced clinical heterogeneity and may have influenced the relative treatment rankings. Drug-specific subgroup analyses were considered; however, they were not statistically feasible because several individual bisphosphonate agents were represented by limited numbers of trials and direct comparisons, which would have resulted in unstable estimates or disconnected networks; (5) a considerable number of included trials had unclear methodological quality, particularly regarding randomization, allocation concealment, and selective outcome reporting. These unclear judgments were mainly attributable to insufficient reporting rather than definite evidence of bias. Nevertheless, the possibility that methodological limitations influenced the pooled estimates and treatment rankings cannot be excluded. Excluding all studies with unclear risk of bias was not methodologically feasible because it would have substantially reduced the available evidence and could have compromised network connectivity. Therefore, the results should be interpreted with caution, especially for comparisons supported by limited or methodologically unclear evidence.

Future research should focus on: (1) extending follow-up durations to evaluate long-term bone mass maintenance and fracture prevention; (2) increasing sample sizes and conducting more direct-comparison studies, especially randomized trials comparing anabolic and antiresorptive agents; (3) exploring individualized treatment strategies, including optimization of drug combinations based on fracture risk, age, and comorbidities; (4) investigating female-specific bone metabolic characteristics and differential responses across fracture sites to guide precision therapy.

## 5 Conclusion

ABA and TER showed favorable efficacy in improving lumbar spine and femoral neck BMD, while ABA ranked highest for total hip BMD in the SUCRA analysis and OBP showed a statistically significant improvement compared with PLA/CTRL. TER and OBP showed relatively favorable ranking probabilities for all AEs, and ABA showed a relatively favorable ranking probability for SAEs. Because the present analysis was mainly based on BMD and safety outcomes, these findings should be interpreted as evidence for site-specific BMD improvement and exploratory safety comparisons rather than direct evidence of comparative fracture-prevention efficacy. Further studies incorporating fracture outcomes and more detailed patient-level characteristics are needed to validate these findings.
