# Supplementary material for: Efficacy and safety of abaloparatide, denosumab, teriparatide, oral bisphosphonates, and intravenous bisphosphonates in the treatment of postmenopausal osteoporosis: a systematic review and Bayesian network meta-analysis
Source: Front Endocrinol (Lausanne). 2026 Jul 9;17:1886136. doi: 10.3389/fendo.2026.1886136 (PMC13392484; doi:10.3389/fendo.2026.1886136)

**Appendix.**

**Supplementary data**

**Search Strategy;**

**PUBMED (1178)**

(((((((((((((((((((((((Osteoporosis[MeSH Terms]) OR (Osteoporoses[Title/Abstract])) OR (Osteoporosis, Age-Related[Title/Abstract])) OR (Osteoporosis, Age Related[Title/Abstract])) OR (Age-Related Osteoporosis[Title/Abstract])) OR (Age-Related Osteoporoses[Title/Abstract])) OR (Age Related Osteoporosis[Title/Abstract])) OR (Osteoporoses, Age-Related[Title/Abstract])) OR (Bone Loss, Age-Related[Title/Abstract])) OR (Age-Related Bone Loss[Title/Abstract])) OR (Age-Related Bone Losses[Title/Abstract])) OR (Bone Loss, Age Related[Title/Abstract])) OR (Bone Losses, Age-Related[Title/Abstract])) OR (Osteoporosis, Senile[Title/Abstract])) OR (Osteoporoses, Senile[Title/Abstract])) OR (Senile Osteoporoses[Title/Abstract])) OR (Senile Osteoporosis[Title/Abstract])) OR (Osteoporosis, Involutional[Title/Abstract])) OR (Osteoporosis, Post-Traumatic[Title/Abstract])) OR (Osteoporosis, Post Traumatic[Title/Abstract])) OR (Post-Traumatic Osteoporoses[Title/Abstract])) OR (Post-Traumatic Osteoporosis[Title/Abstract])) AND (Women[MeSH Terms] OR Female[MeSH Terms] OR Females[Title/Abstract] OR Postmenopausal[Title/Abstract]) AND (((((((((((((((((((((((((((((((((((((Diphosphonates[MeSH Terms]) OR (Bisphosphonate[Title/Abstract])) OR (Bisphosphonates[Title/Abstract])) OR (Alendronate[MeSH Terms])) OR (Aminohydroxybutane Bisphosphonate[Title/Abstract])) OR (4-Amino-1-Hydroxybutylidene 1,1-Biphosphonate[Title/Abstract])) OR (MK-217[Title/Abstract])) OR (MK217[Title/Abstract])) OR (MK 217[Title/Abstract])) OR (Fosamax[Title/Abstract])) OR (Alendronate Sodium[Title/Abstract])) OR (Alendronate Monosodium Salt, Trihydrate[Title/Abstract])) OR (Risedronic Acid[MeSH Terms])) OR (Bisphosphonate Risedronate Sodium[Title/Abstract])) OR (Risedronate Sodium, Bisphosphonate[Title/Abstract])) OR (Sodium, Bisphosphonate Risedronate[Title/Abstract])) OR (Risedronate Sodium[Title/Abstract])) OR (Ibandronic Acid[MeSH Terms])) OR (Ibandronate[Title/Abstract])) OR ((1-Hydroxy-3-(methylpentylamino)propylidene)bisphosphonate[Title/Abstract])) OR (1-Hydroxy-3-(methylpentylamino)propylidenebisphosphonate[Title/Abstract])) OR (abaloparatide[MeSH Terms])) OR (BA058[Title/Abstract])) OR (Tymlos[Title/Abstract])) OR (Denosumab[MeSH Terms])) OR (AMG 162[Title/Abstract])) OR (Xgeva[Title/Abstract])) OR (Prolia[Title/Abstract])) OR (Teriparatide[MeSH Terms])) OR (hPTH (1-34[Title/Abstract]))) OR (Human Parathyroid Hormone (1-34[Title/Abstract]))) OR (Parathar[Title/Abstract])) OR (Teriparatide Acetate[Title/Abstract])) OR (Forteo[Title/Abstract])) OR (Zoledronic Acid[MeSH Terms])) OR (2-(Imidazol-1-yl)-1-hydroxyethylidene-1,1-bisphosphonic acid[MeSH Terms])) OR (Zoledronic Acid Anhydrous[MeSH Terms]))AND(Randomized Controlled Trial[Publication Type] OR Randomized[Title/Abstract] OR Randomised[Title/Abstract] OR RCT[Title/Abstract] OR Clinical Trial[Title/Abstract])

**Cochrane Central Register of Controlled Trials (1437)**

#1 MeSH descriptor: [Osteoporosis] explode all trees

#2 MeSH descriptor: [Women] explode all trees

#3 MeSH descriptor: [Female] explode all trees

#4 #2 OR #3

#5 #1 AND #4

#6 MeSH descriptor: [Diphosphonates] explode all trees

#7 MeSH descriptor: [Alendronate] explode all trees

#8 MeSH descriptor: [Risedronic Acid] explode all trees

#9 MeSH descriptor: [Ibandronic Acid] explode all trees

#10 MeSH descriptor: [Denosumab] explode all trees

#11 MeSH descriptor: [Teriparatide] explode all trees

#12 MeSH descriptor: [Zoledronic Acid] explode all trees

#13 #6 OR #7 OR #8 OR #9 OR #10 OR #11 OR #12

#14 #5 AND #13 1437

**Web of science (2901)**

1: (((((((((((((((((TS=(Osteoporosis)) OR TS=(Osteoporoses)) OR TS=(Osteoporosis, Age-Related)) OR TS=(Osteoporosis, Age Related)) OR TS=(Age-Related Osteoporosis)) OR TS=(Age-Related Osteoporoses)) OR TS=(Age Related Osteoporosis)) OR TS=(Osteoporoses, Age-Related)) OR TS=(Bone Loss, Age-Related)) OR TS=(Age-Related Bone Loss)) OR TS=(Age-Related Bone Losses)) OR TS=(Bone Loss, Age Related)) OR TS=(Bone Losses, Age-Related)) OR TS=(Osteoporosis, Senile)) OR TS=(Osteoporoses, Senile)) OR TS=(Senile Osteoporoses)) OR TS=(Senile Osteoporosis)) OR TS=(Osteoporosis, Involutional)

2: TS=(Women) OR TS=(Female) OR TS=(Females) OR TS=(Postmenopausal)

3: (((((((((((((((((((((((((((((TS=(Diphosphonates)) OR TS=(Bisphosphonate)) OR TS=(Bisphosphonates)) OR TS=(Alendronate)) OR TS=(Aminohydroxybutane Bisphosphonate)) OR TS=(4-Amino-1-Hydroxybutylidene 1,1-Biphosphonate)) OR TS=(Alendronate Sodium)) OR TS=(Alendronate Monosodium Salt, Trihydrate)) OR TS=(Risedronic Acid)) OR TS=(Bisphosphonate Risedronate Sodium)) OR TS=(Risedronate Sodium, Bisphosphonate)) OR TS=(Sodium, Bisphosphonate Risedronate)) OR TS=(Risedronate Sodium)) OR TS=(Ibandronic Acid)) OR TS=(Ibandronate)) OR TS=((1-Hydroxy-3-(methylpentylamino)propylidene)bisphosphonate)) OR TS=(1-Hydroxy-3-(methylpentylamino)propylidenebisphosphonate)) OR TS=(abaloparatide )) OR TS=(Tymlos)) OR TS=(Denosumab)) OR TS=(Xgeva)) OR TS=(Prolia)) OR TS=(Teriparatide)) OR TS=(hPTH (1-34))) OR TS=(Human Parathyroid Hormone (1-34))) OR TS=(Parathar)) OR TS=(Teriparatide Acetate)) OR TS=(Zoledronic Acid)) OR TS=(2-(Imidazol-1-yl)-1-hydroxyethylidene-1,1-bisphosphonic acid)) OR TS=(Zoledronic Acid Anhydrous)

4: ((((TS=(randomized controlled trial)) OR TS=(randomized )) OR TS=(Clinical Trials, Randomized)) OR TS=(Trials, Randomized Clinical)) OR TS=(Controlled Clinical Trials, Randomized)

5: #1 AND #2 AND #3 AND #4

**Supplementary Table 1.** Model Fit Assessment: If the difference in DIC values between two models is within 5, it indicates that the data is consistent. DIC stands for Deviance Information Criterion.

|  | The Global inconsistency | ＞0.05 | DIC of Model of consistency | DIC of Model of inconsistency | The difference is  less than 5 |
| --- | --- | --- | --- | --- | --- |
| Lumbar spine BMD | 0.0747 | yes | 36.12561 | 39.12565 | yes |
| Femoral neck BMD | 0.0674 | yes | 40.45789 | 36.45677 | yes |
| Total hip BMD | 0.0848 | yes | 35.34572 | 34.154336 | yes |
| All adverse events | 0.3653 | yes | 35.56892 | 39.12334 | yes |
| Serious adverse events | 0.4353 | yes | 26.15689 | 22.56829 | yes |

**Supplementary Figure 1.** The [forest map](javascript:;) of all outcomes.

a)The results of [forest map](javascript:;) for Lumbar spine BMD.


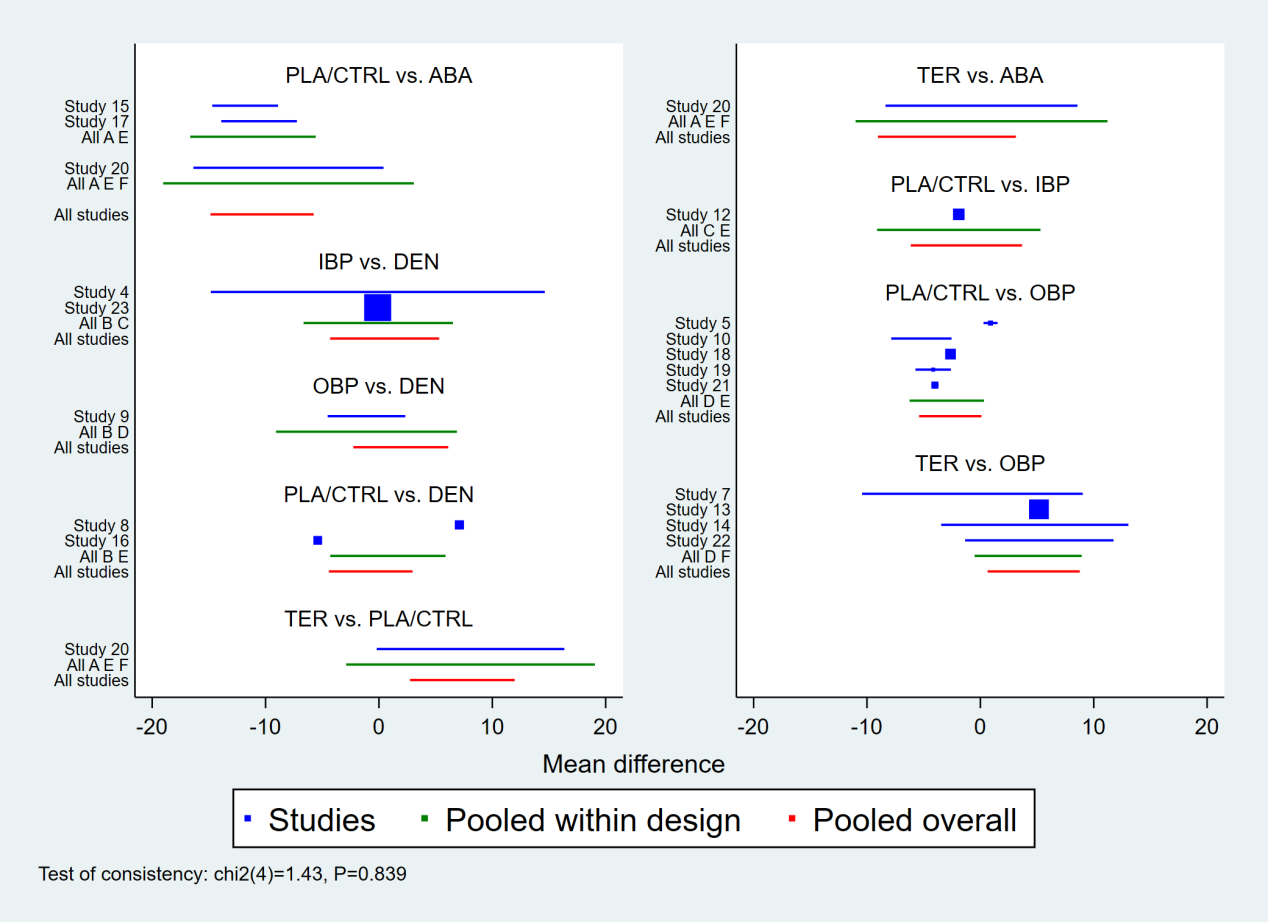


b) The results of [forest map](javascript:;) for Femoral neck BMD.


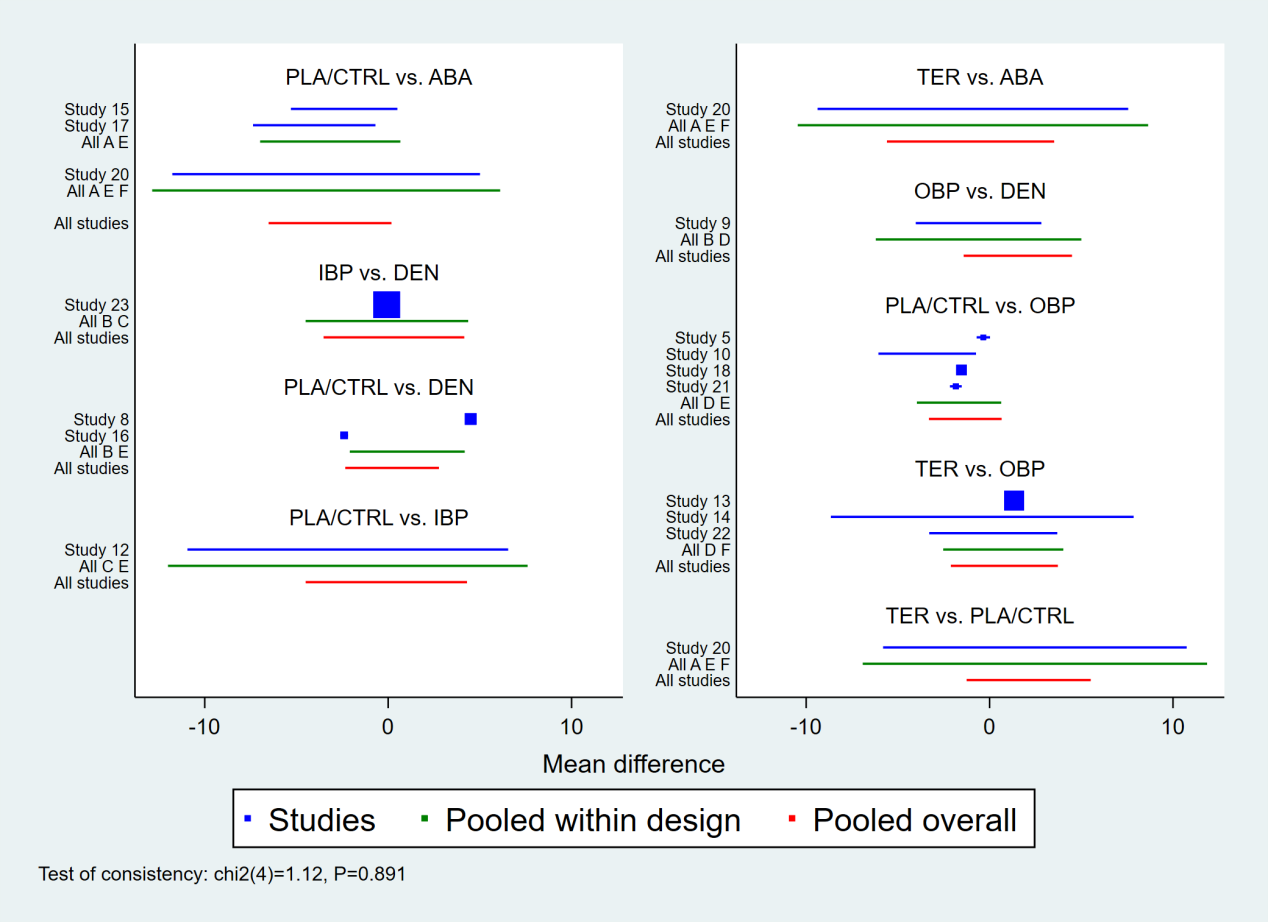


c) The results of [forest map](javascript:;) for Total hip BMD.


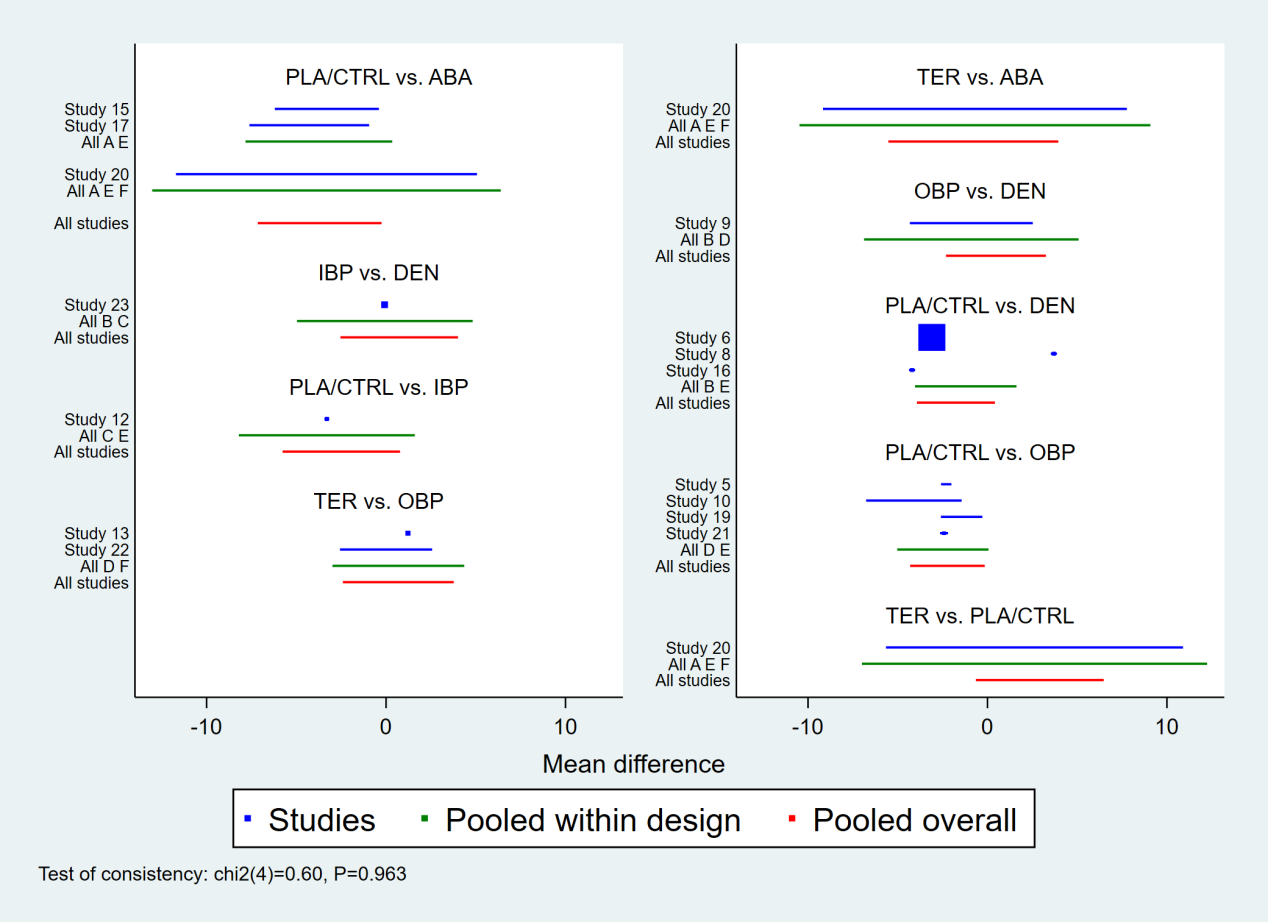


d) The results of [forest map](javascript:;) for All adverse events.


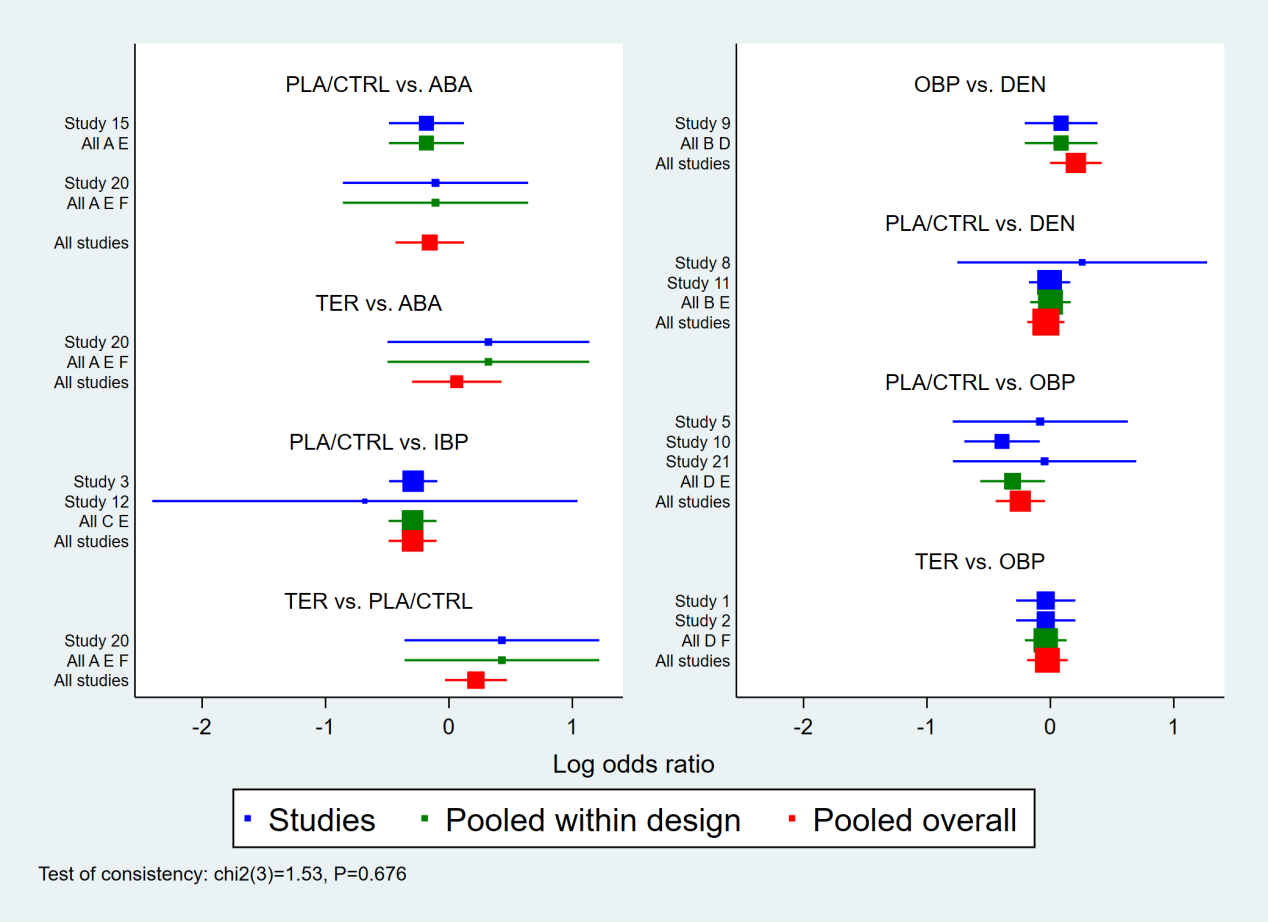


e) The results of [forest map](javascript:;) for Serious adverse events.


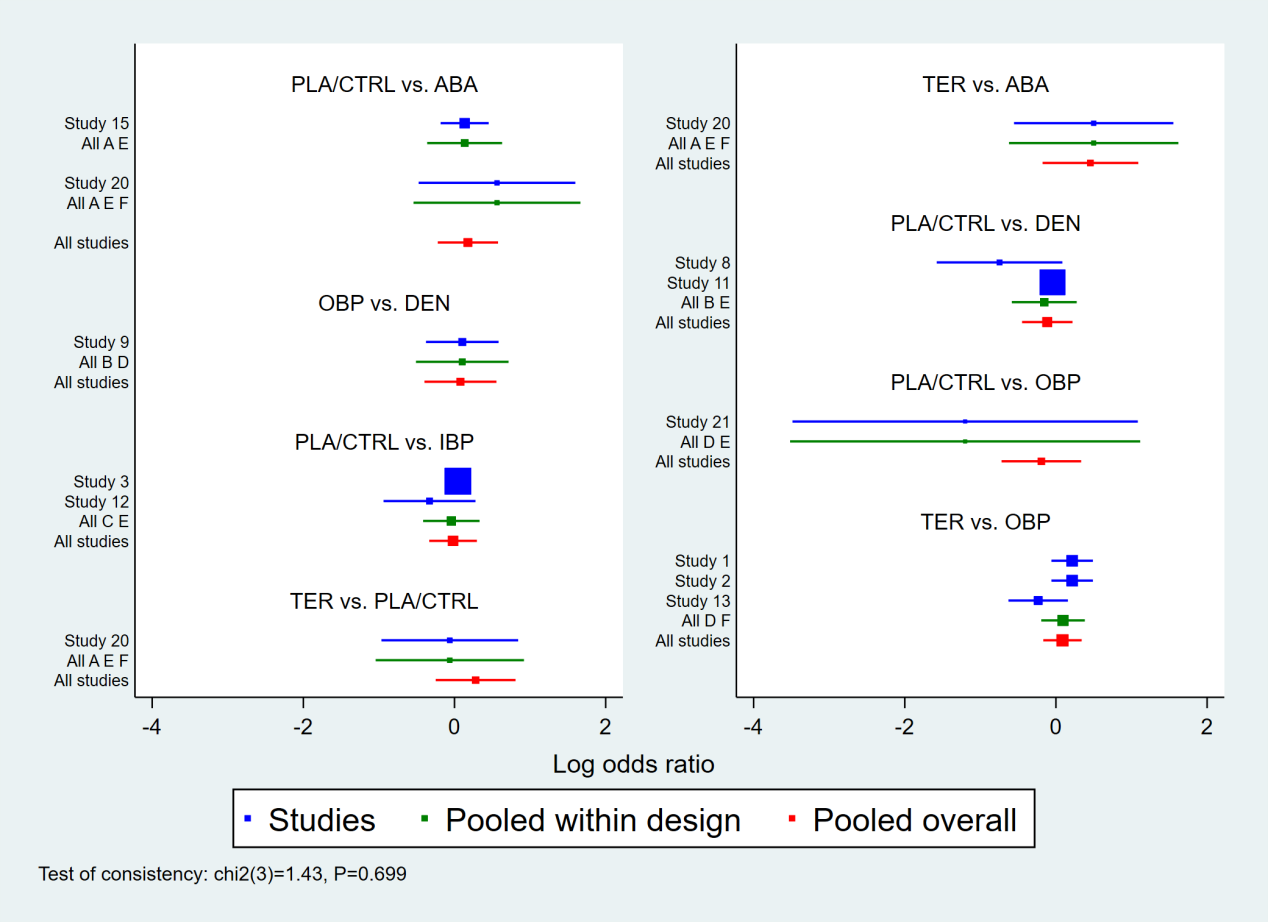


**Supplementary Figure 2.** The network plot of all outcomes.

1. Lumbar spine BMD


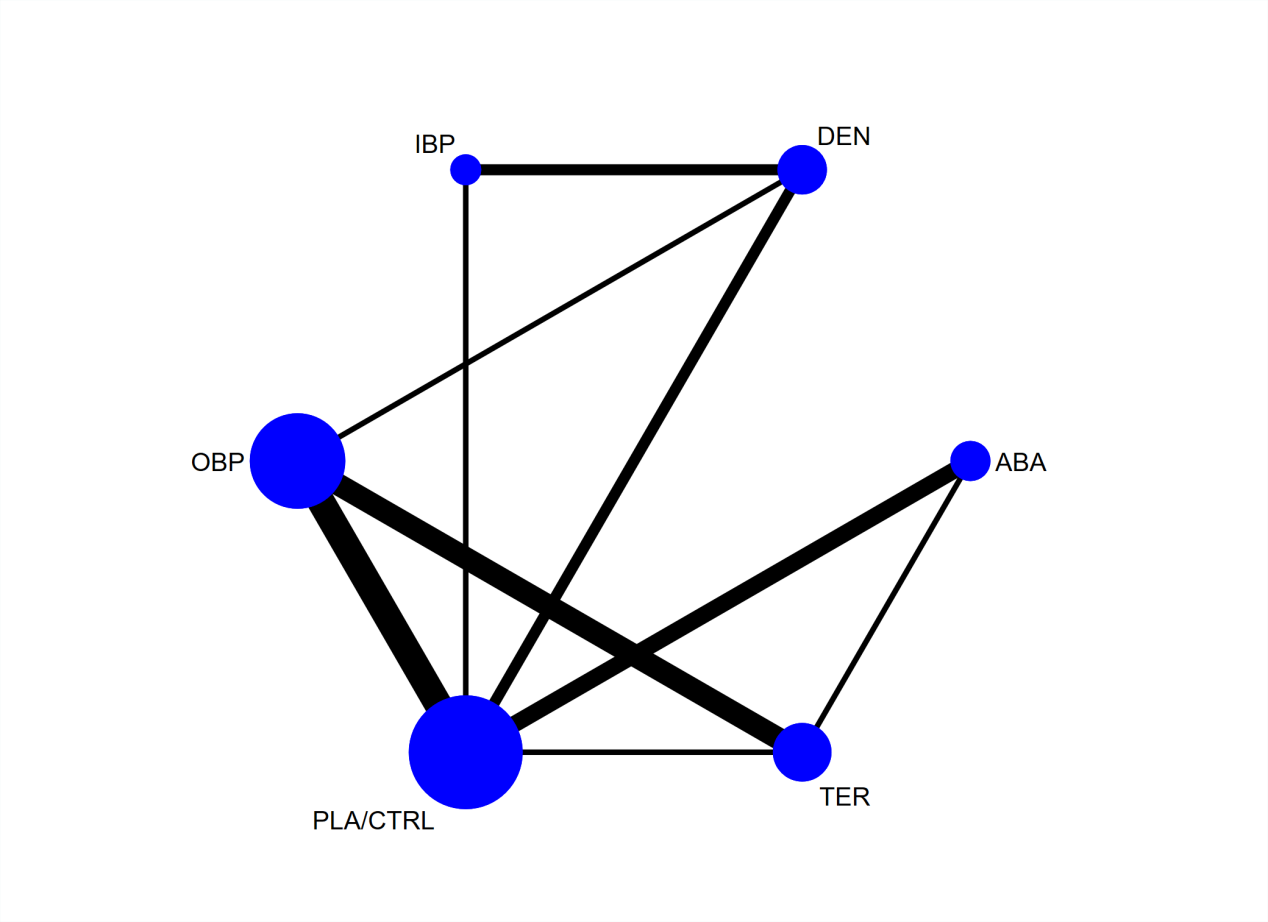


1. Femoral neck BMD


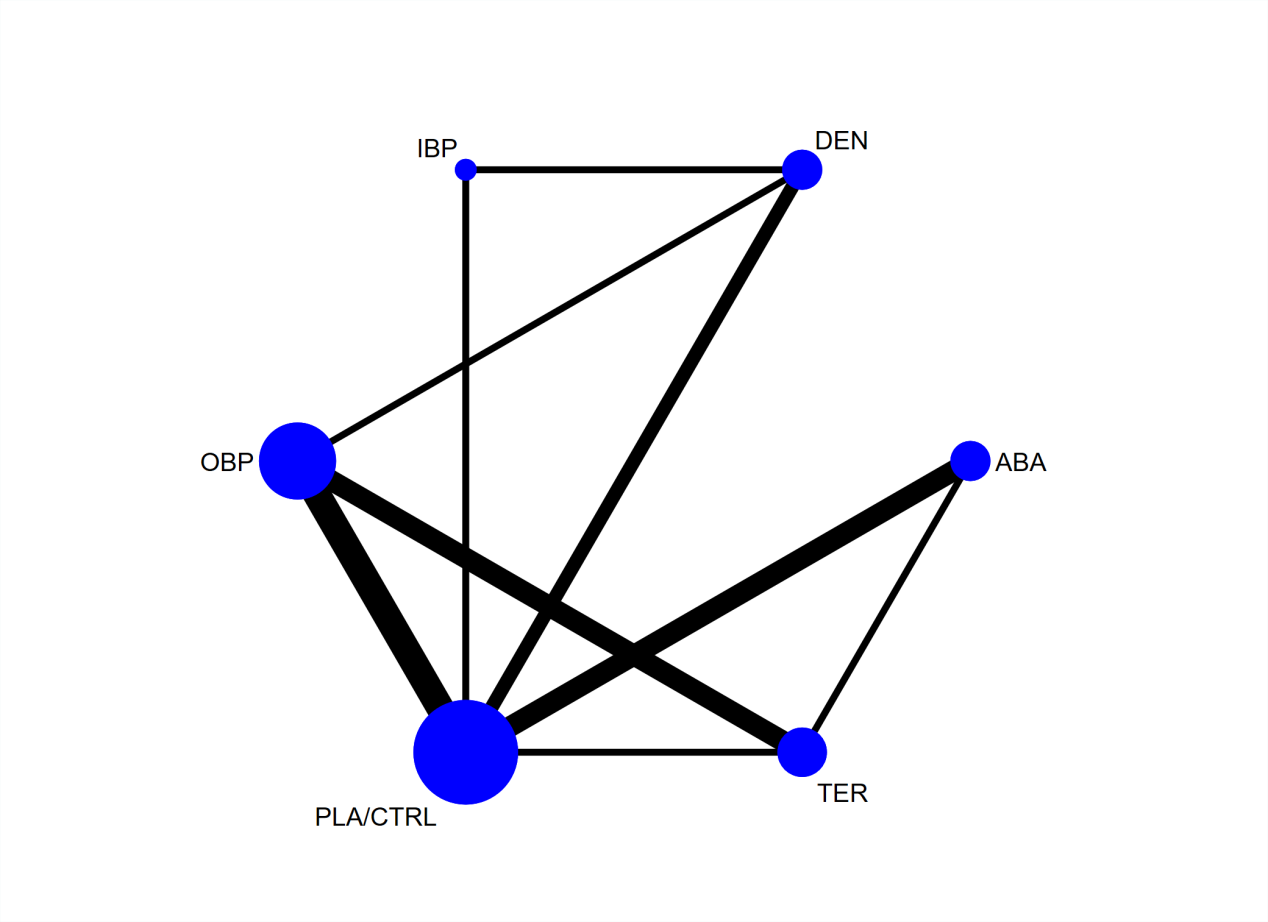


1. Total hip BMD


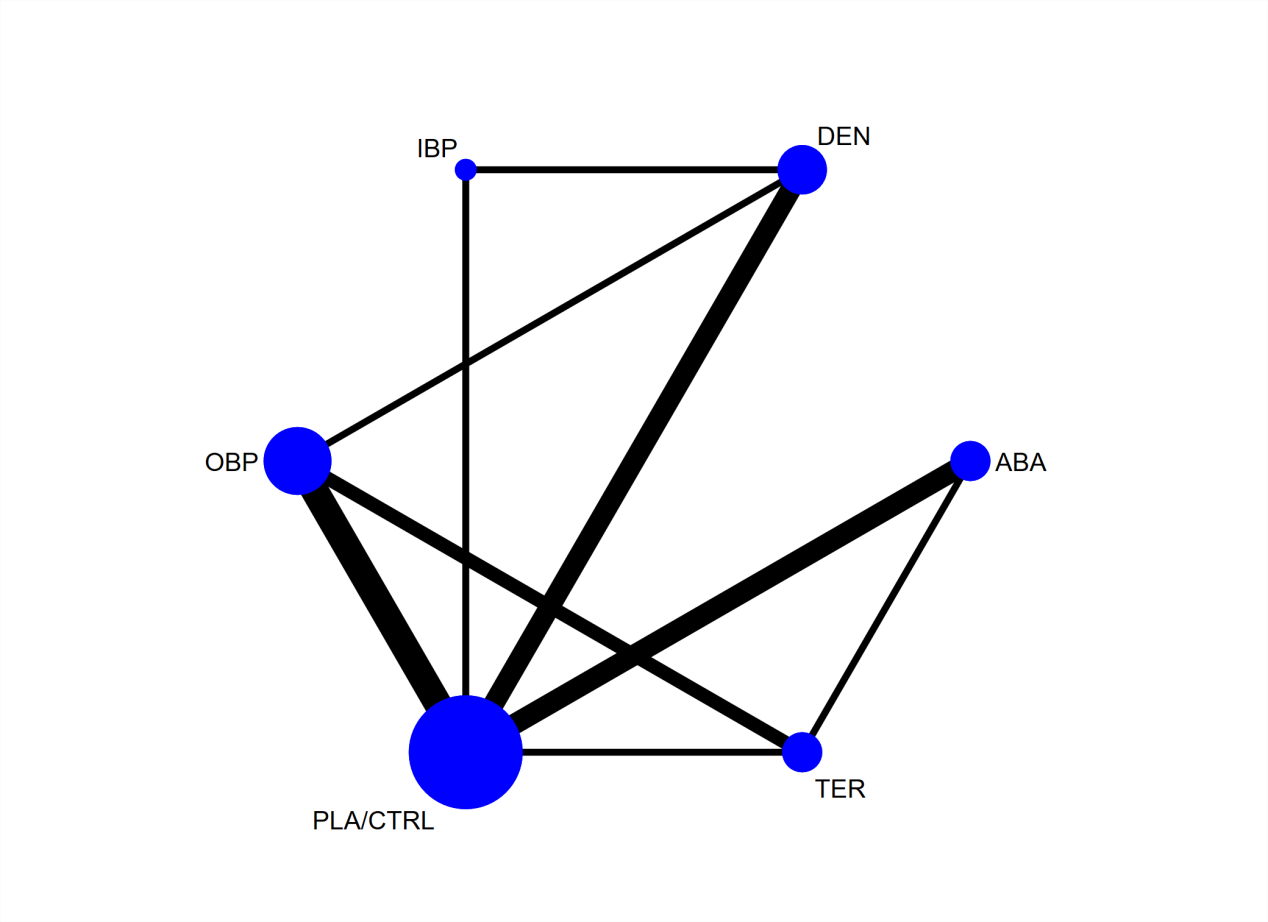


1. All adverse events


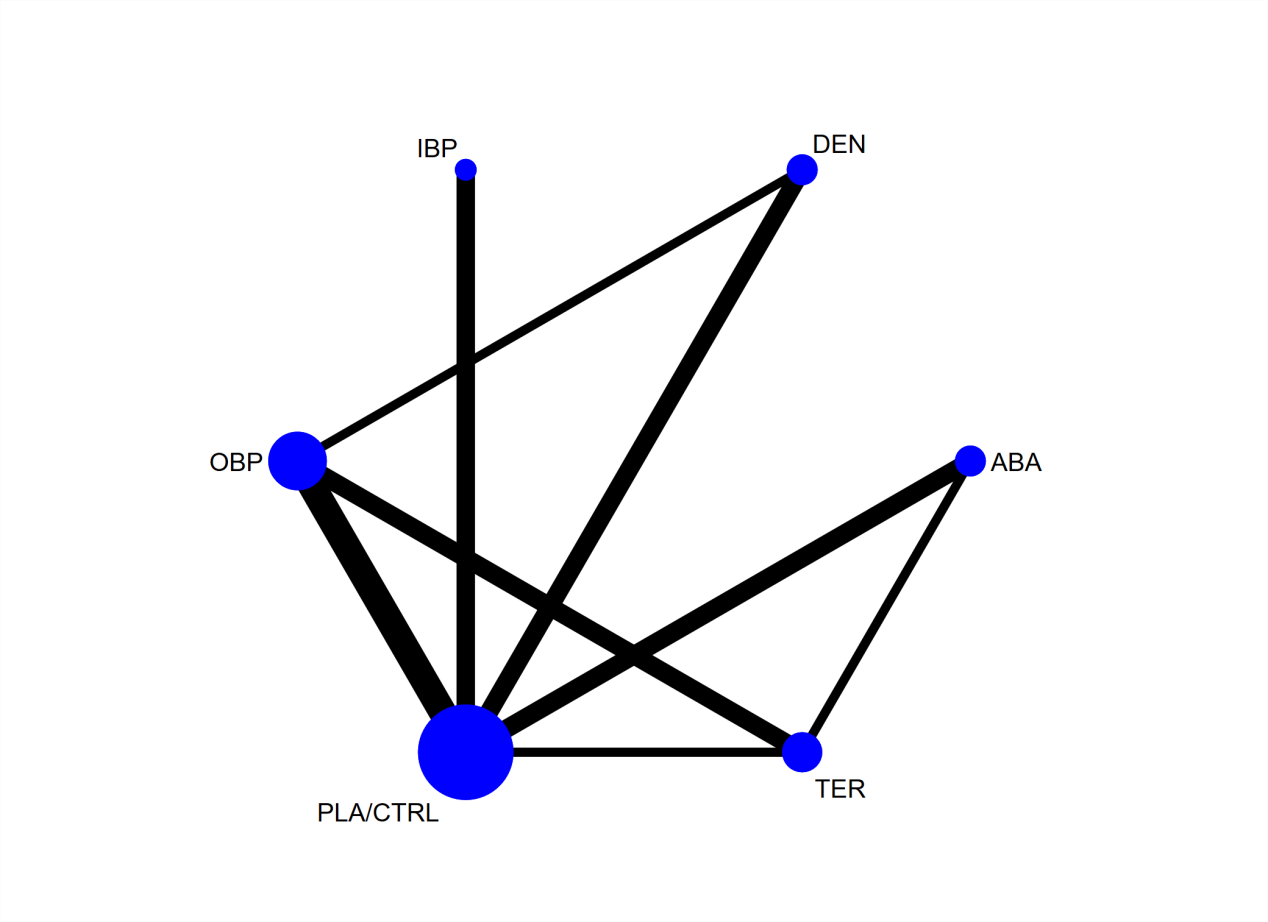


1. Serious adverse events


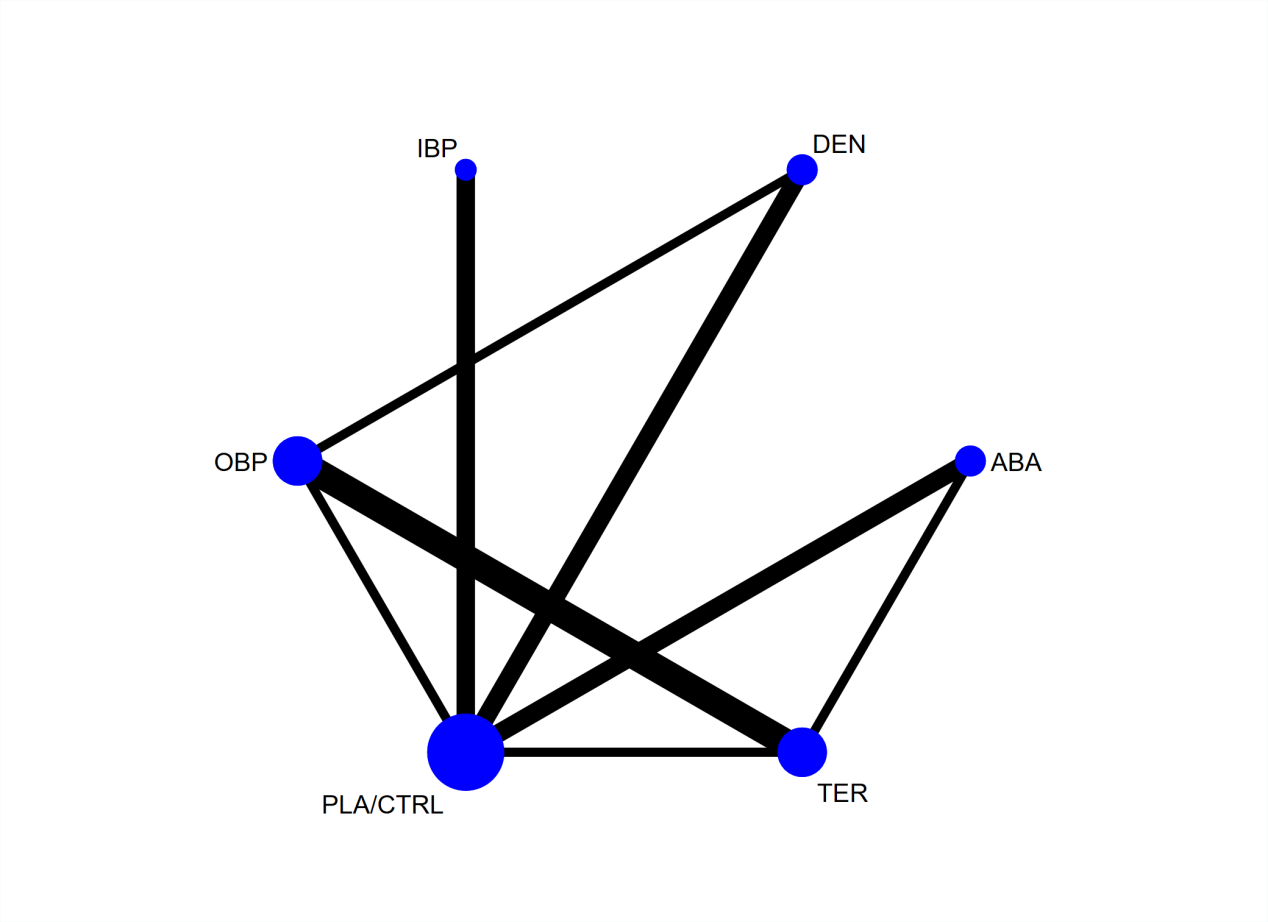


**Supplementary Figure 3.** The Funnel plot of all outcomes.

1. Lumbar spine BMD


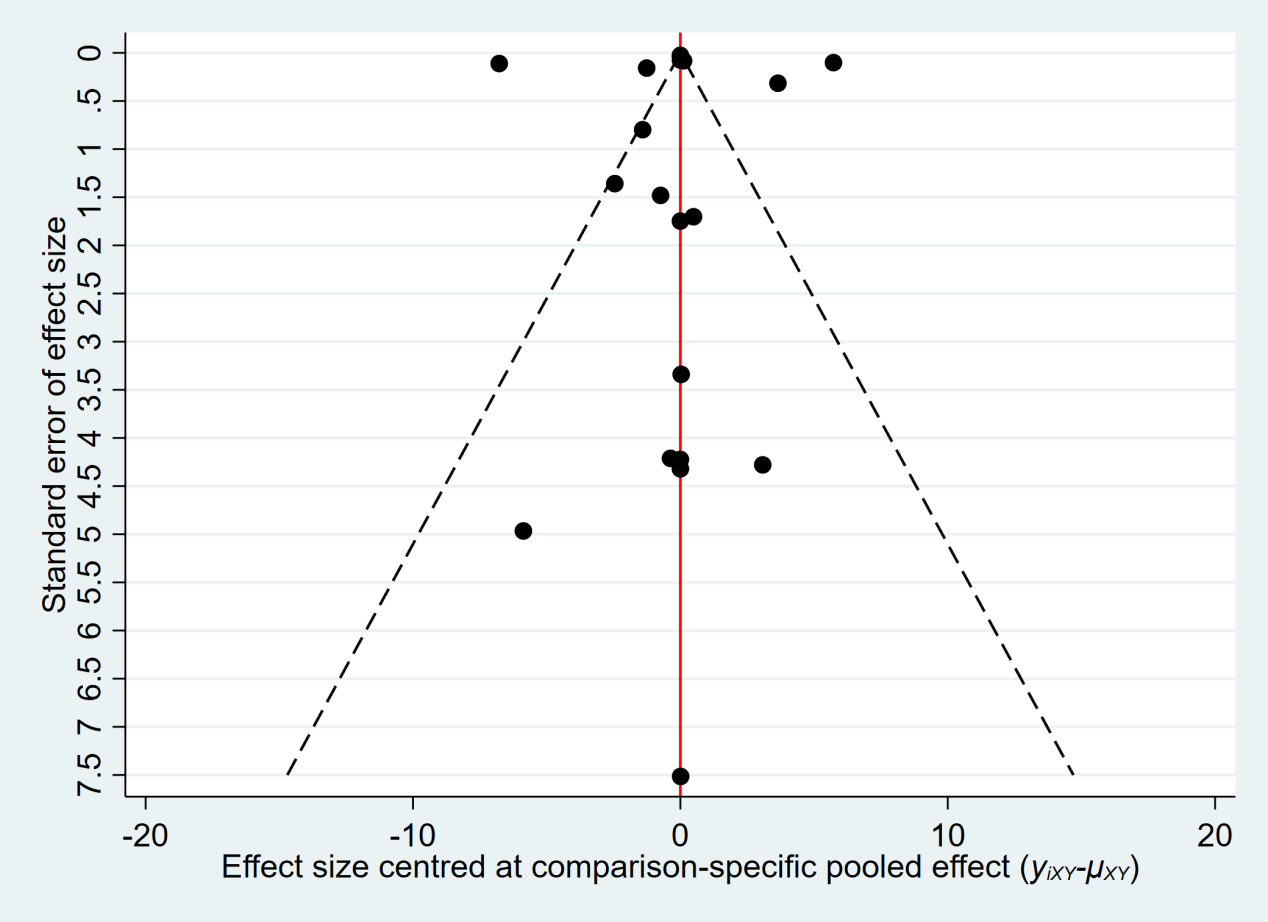


1. Femoral neck BMD


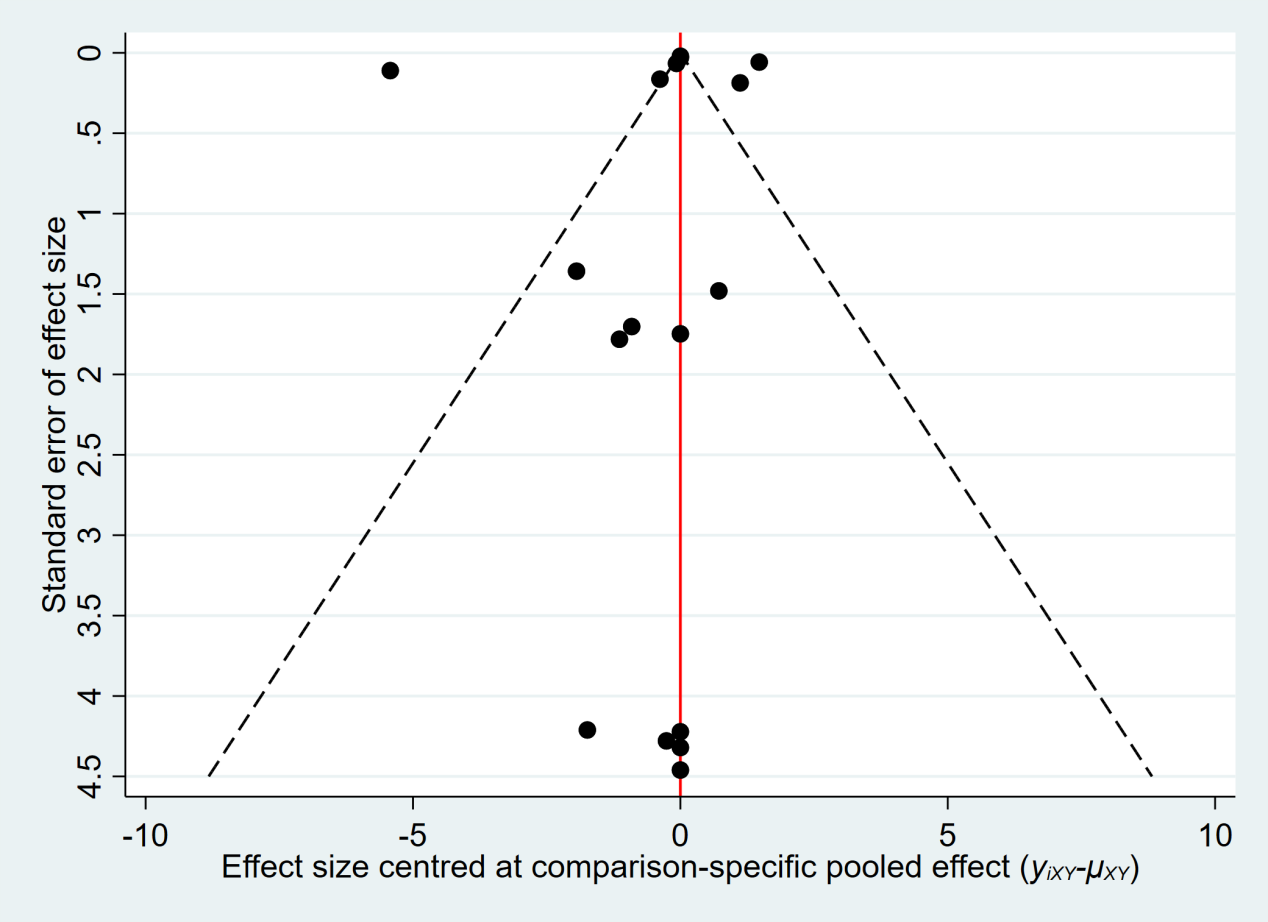


1. Total hip BMD


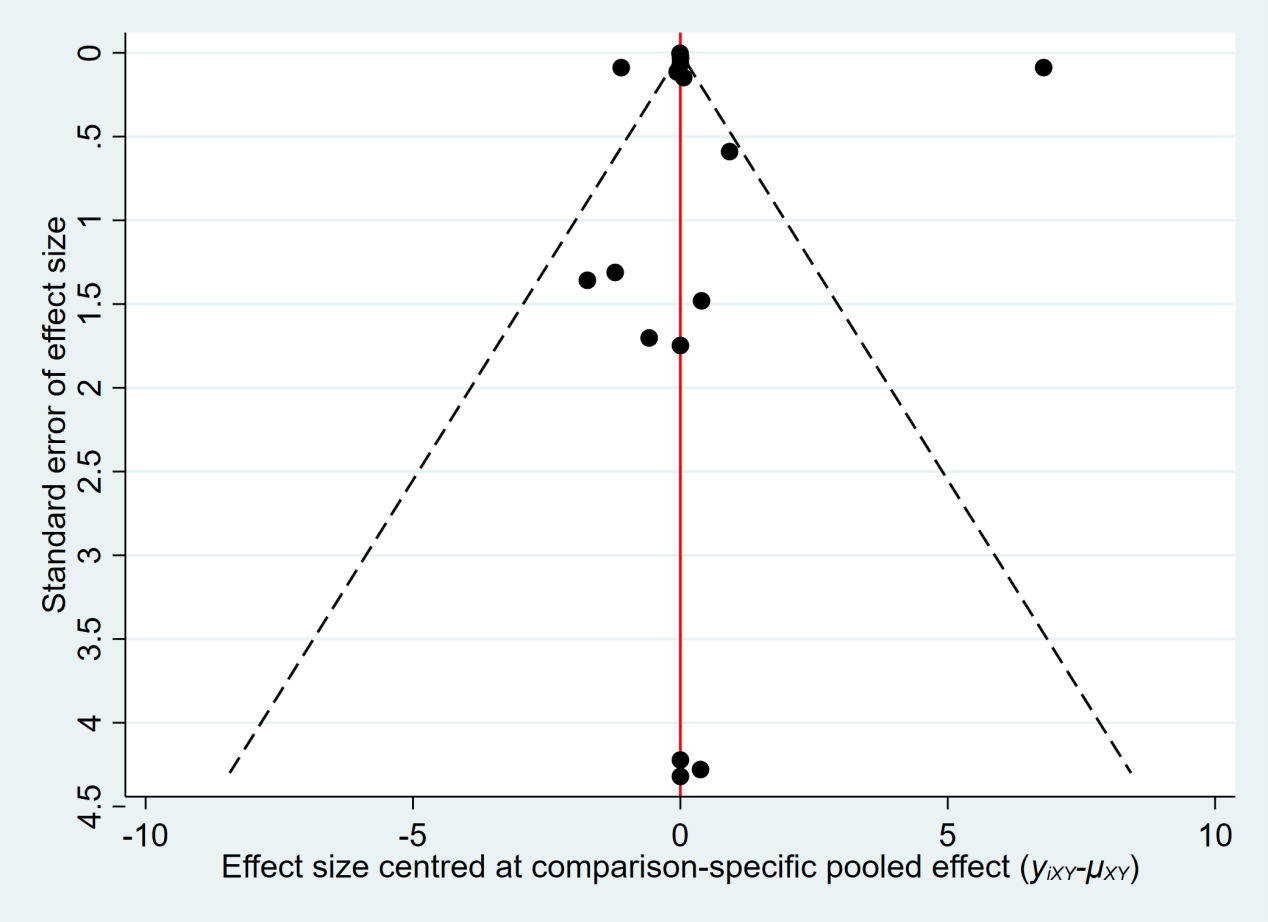


1. All adverse events


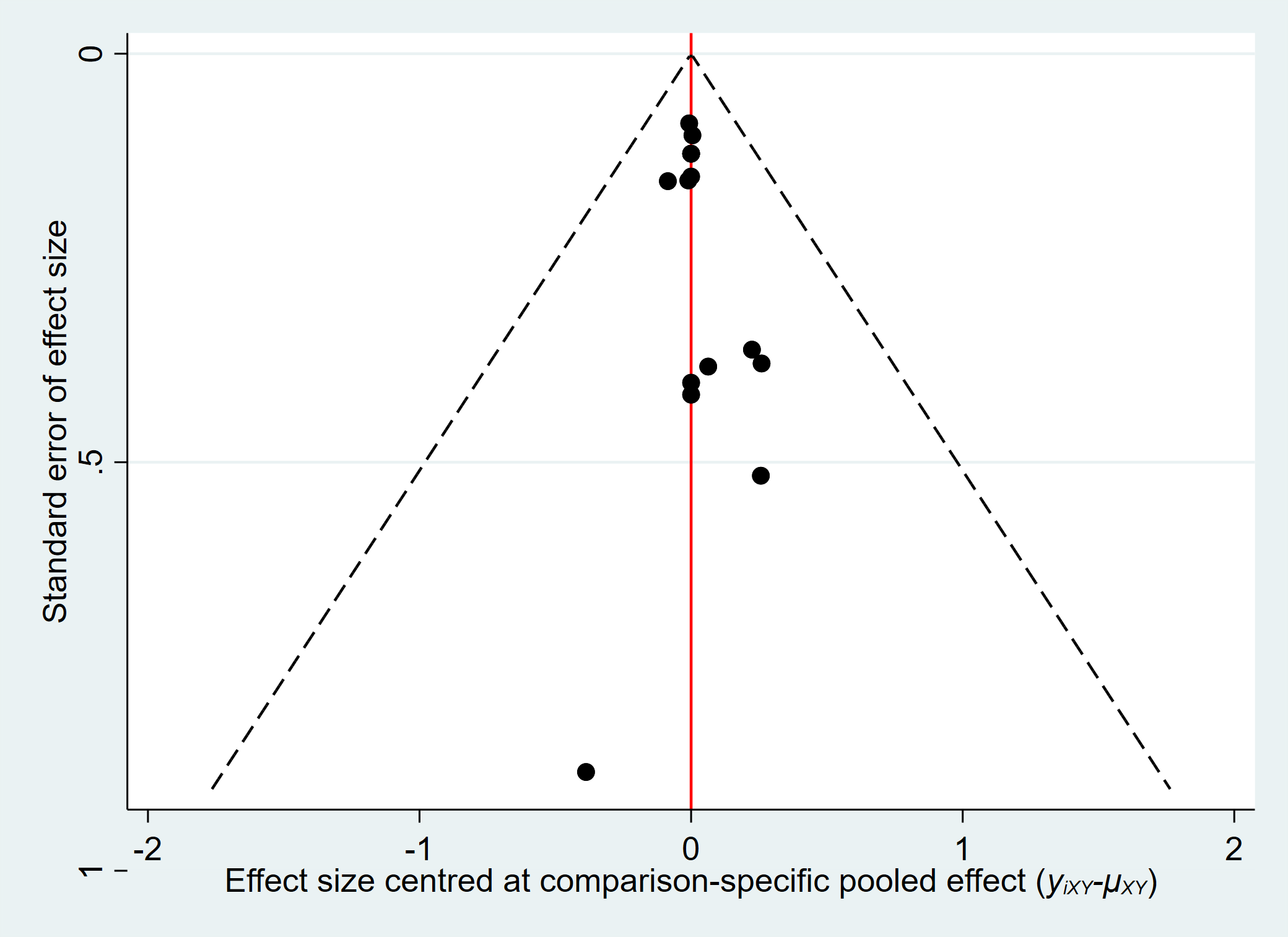


1. Serious adverse events


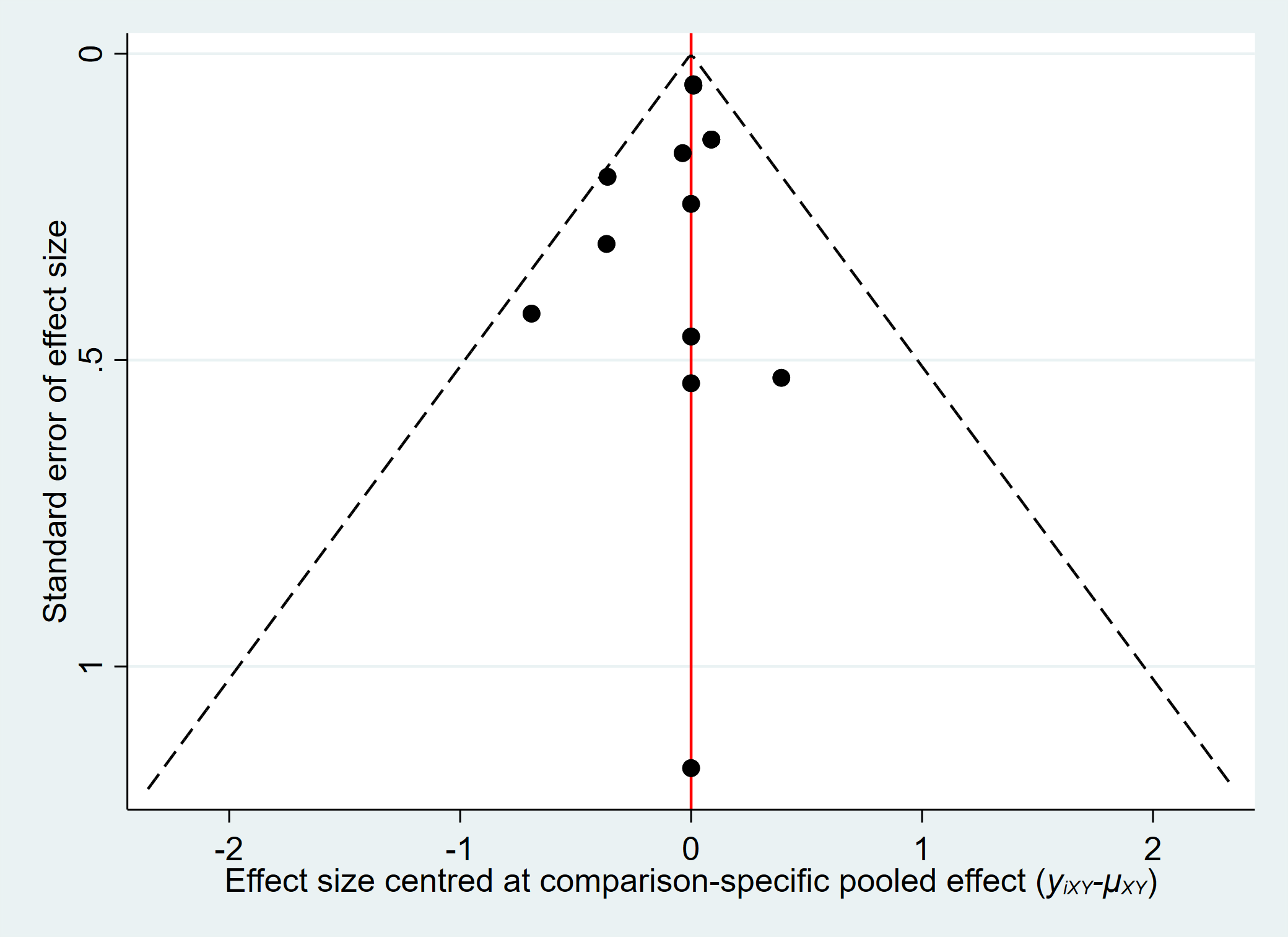

Supplement: Supplementary file 1 [file DataSheet1.docx]
